# Interferon-Gamma at the Crossroads of Tumor Immune Surveillance or Evasion

**DOI:** 10.3389/fimmu.2018.00847

**Published:** 2018-05-04

**Authors:** Flávia Castro, Ana Patrícia Cardoso, Raquel Madeira Gonçalves, Karine Serre, Maria José Oliveira

**Affiliations:** ^1^i3S – Instituto de Investigação e Inovação em Saúde, Universidade do Porto, Porto, Portugal; ^2^INEB – Instituto de Engenharia Biomédica, Universidade do Porto, Porto, Portugal; ^3^ICBAS – Instituto de Ciências Biomédicas Abel Salazar, Universidade do Porto, Porto, Portugal; ^4^IMM – Instituto de Medicina Molecular João Lobo Antunes, Faculdade de Medicina, Universidade de Lisboa, Lisbon, Portugal; ^5^Departamento de Patologia e Oncologia, Faculdade de Medicina, Universidade do Porto, Porto, Portugal

**Keywords:** type II interferon, immunoregulation, cancer microenvironment, immunotherapy, immune contexture

## Abstract

Interferon-gamma (IFN-γ) is a pleiotropic molecule with associated antiproliferative, pro-apoptotic and antitumor mechanisms. This effector cytokine, often considered as a major effector of immunity, has been used in the treatment of several diseases, despite its adverse effects. Although broad evidence implicating IFN-γ in tumor immune surveillance, IFN-γ-based therapies undergoing clinical trials have been of limited success. In fact, recent reports suggested that it may also play a protumorigenic role, namely, through IFN-γ signaling insensitivity, downregulation of major histocompatibility complexes, and upregulation of indoleamine 2,3-dioxygenase and of checkpoint inhibitors, as programmed cell-death ligand 1. However, the IFN-γ-mediated responses are still positively associated with patient’s survival in several cancers. Consequently, major research efforts are required to understand the immune contexture in which IFN-γ induces its intricate and highly regulated effects in the tumor microenvironment. This review discusses the current knowledge on the pro- and antitumorigenic effects of IFN-γ as part of the complex immune response to cancer, highlighting the relevance to identify IFN-γ responsive patients for the improvement of therapies that exploit associated signaling pathways.

## Introduction

Interferons (IFNs) are pleiotropic cytokines with antiviral, antitumor and immunomodulatory properties, being central coordinators of the immune response (1). The term “interferons” comes from the description of molecules protecting cells by “interfering” with viral infection (2, 3). Three major types of IFNs are distinguished by their sequence identity, genetic loci, cell of origin, nature, and distribution of their receptors and resulting stimuli (Table 1).

**Table 1:** Comparison of human type I, type II, and type III IFN production and signaling.

Properties	Type I IFN (IFN-α, IFN-β)	Type II IFN (IFN-γ)	Type III IFN (IFN-λ)
Members	17 proteins: 13 IFN-α, IFN-β, IFN-ε, IFN-κ, IFN-ω	1 protein: IFN-γ	4 proteins: IFN-λ1, IFN-λ2, IFN-λ3, IFN-λ4
IFN-producing cells	All nucleated cells	T cells, B cells, NK cells, NKT cells, and APCs	All nucleated cells, mainly mDCs, pDCs, and epithelial cells
IFN-responding cells	All nucleated cells	All nucleated cells	Lung, intestine, and liver epithelial cells
Stimuli	DAMPs and PAMPs	IL-12, IL-15, IL-18, type I IFN, and PAMPs	DAMPs and PAMPs
IFN receptor	IFN type I receptor (IFNαR): IFNαR1 and IFNαR2 subunits	IFN type II receptor (IFNγR): IFNγR1 and IFNγR2 subunits	IFN type III receptor (IFNλR): IFNλR1 and IL10Rβ
Signaling molecules	TYK2, JAK1, all STATs, CRKL, and IRS	JAK1, JAK2, STAT1, and STAT3	TYK2, JAK1, STAT1, STAT2, and IRF9
Transcription factor binding sites	ISRE (canonical) GAS (non-canonical)	GAS (canonical) ISRE (non-canonical)	ISRE
Functions	Antiviral, antiproliferative response, regulation of cell survival/apoptosis, and immunoregulation	Antiviral, antiproliferative, immunomodulatory, and antitumor response	Antiviral response, mucosal immunity
Reference	(4, 5)	(6, 7)	(8)

*APCs, antigen-presenting cells; CRKL, CT10 regulator of kinase-like; DAMPs, damage-associated molecular patterns; GAS, gamma-activated site; IFN, interferon; IFN-γ, interferon-gamma; IFNαR1–2, type I receptor; IFNγR, type II receptor; IFNλR, type III receptor; IL, interleukin; IRF, interferon-regulatory factor; IRS, insulin receptor substrate; ISRE, interferon-sensitive response element; JAK, Janus kinase; mDCs, myeloid dendritic cells; NK, natural killer; NKT, natural killer T cells; PAMPs, pathogen-associated molecular patterns; pDCs, plasmacytoid dendritic cells; STAT, signal transducer and activator of transcription; TYK, tyrosine kinase*.

The human type I IFN family comprises 17 distinct proteins, mainly represented by IFN-α and IFN-β, which are ubiquitously expressed and signal through their cognate receptor, composed by IFNαR1 and IFNαR2 subunits [reviewed in Ref. (4)]. IFN-γ is the lone member of type II IFN family. It is more restrictively expressed and is structurally and functionally different from the other types of IFNs. Most recently, a type III IFN family was described to be composed of four homologous proteins (IFNλ1–4), which bind the IFNλR1 and interleukin (IL)-10Rβ heterodimeric receptor [reviewed in Ref. (8)]. To date, type I and type III IFNs have been mainly involved in host–pathogen interactions, and their expression is activated through immune system sentinel receptors, such as pattern recognition receptors. Despite the similar function of type I and III on antiviral infections, it is the viral tropism that dictates the relative contribution of each IFN (9). Moreover, whereas almost all nucleated cells respond to type I IFN, type III IFNs response is restricted to tissues with a high risk of viral exposure and infection, as the mucosal surfaces. The role of type II IFN in promoting host immune response to microorganisms is similarly well documented. Notably, it is also known to play a pivotal function on cancer immune surveillance, stimulating antitumor immunity and promoting tumor recognition and elimination (10–16).

This review focuses on type II IFN signaling, cellular functions, and directed therapies and was encouraged by novel findings revealing regulatory mechanisms of IFN-γ and its prognostic as well as therapeutic potential. In fact, since Wheelock who reported that IFN-γ inhibited viral replication in 1965 (17), it took around 30 years to envisage this cytokine as a target of antitumor immunity (18).

Interferon-gamma is a homodimer formed by the non-covalent association of two 17 kDa polypeptide subunits. During synthesis, after multiple N-glycosylation, both subunits bind in an antiparallel manner, constituting a mature 50 kDa molecule (19, 20). Notably, the IFN-γ symmetry suggests that a single molecule can bind simultaneously to two receptors, amplifying the underlying responses. Cellular responses induced by IFN-γ may also involve cross-communication with IFN-α/β receptors, amplifying IFN-γ signaling and its effects (21, 22).

Interferon-gamma is secreted predominantly by activated lymphocytes such as CD4 T helper type 1 (Th1) cells and CD8 cytotoxic T cells (23–26), γδ T cells (27–33), and natural killer (NK) cells (34, 35) and, to a less extent, by natural killer T cells (NKT), B cells (36–39), and professional antigen-presenting cells (APCs) (40–42). Its expression is induced by mitogens and cytokines, such as IL-12 (43, 44), IL-15 (45), IL-18 (46, 47), and type I IFN (48, 49). IFN-γ pleiotropic functions are mediated by cell-specific expression of hundreds of IFN-γ-regulated genes that encompass inflammatory signaling molecules, apoptosis and cell cycle regulators, and transcriptional activators (50). Autocrine IFN-γ produced by APCs can act locally and contribute to sustain self and neighbor cell activation (51–53), crucial for early control of pathogen spreading, while T lymphocytes are the major paracrine source of IFN-γ in adaptive immunity. Under physiological conditions, the constitutive expression of type I and II IFNs is tightly controlled, remaining localized to tissues, without systemic effects (54–56). For instance, constitutive expression of endogenous IFN-γ contributes to the homeostasis of immune cell functions (57), maintenance of the hematopoietic stem cell niche (58), and bone formation (59). Combination approaches to boost innate immune activation have been explored to converge onto IFN pathways. However, IFN-γ-related signaling can also have suppressive immunoregulatory effects on antiviral (60, 61), autoimmune (62, 63), as well as on antitumor responses (64, 65). Unveiling cellular targets of IFN-γ is critically important for its therapeutic application, to predict patient responses, particularly in cancers where this cytokine can exert protumorigenic effects. Therefore, the cellular and molecular effects of IFN-γ, with particular emphasis on its dual role on tumor immunity and how to overcome its limitations, will be the major focus of this review.

## Canonical Signaling and Regulatory Mechanisms

### The IFN-γ Receptor

**Figure 1:**
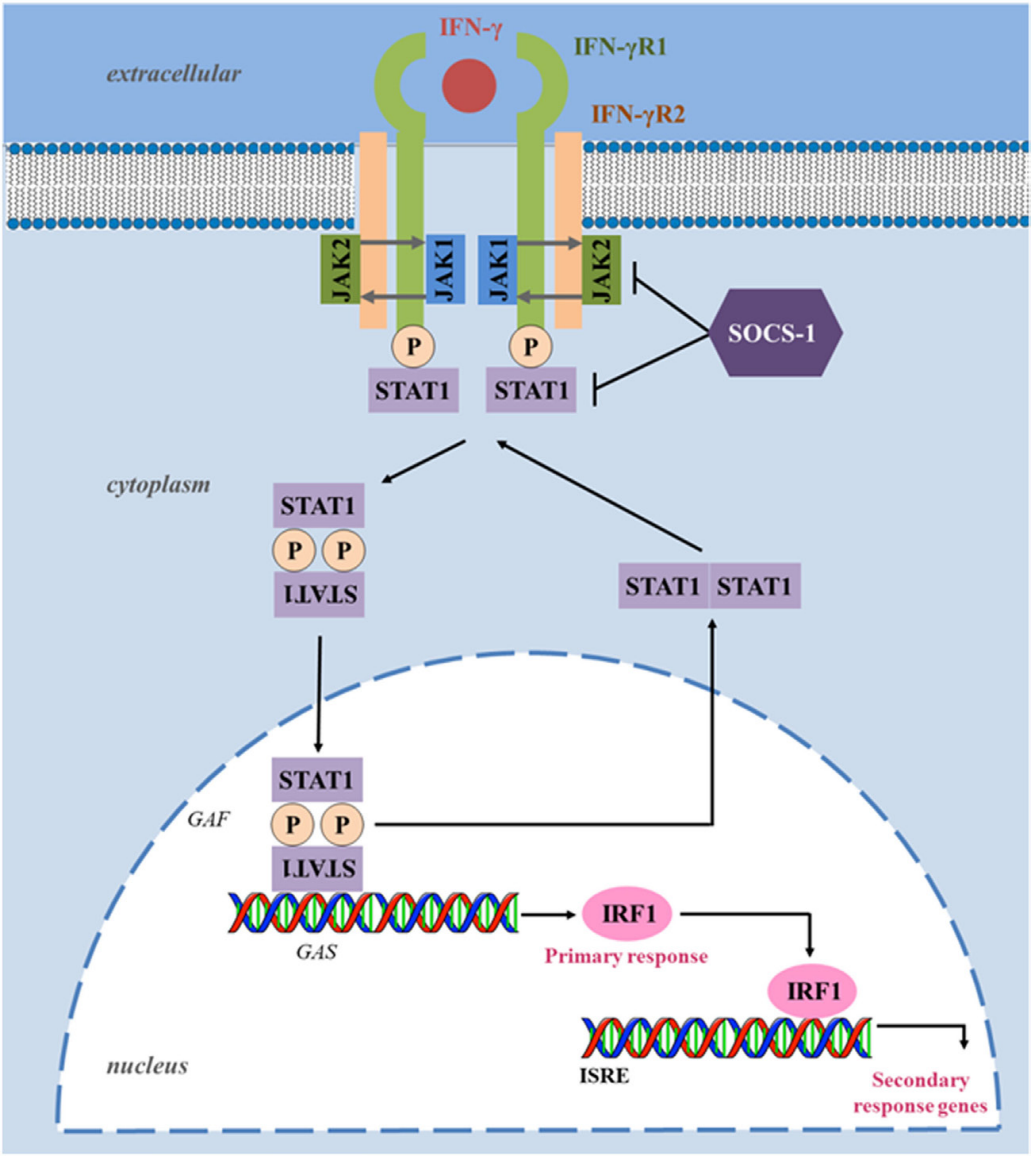
Interferon-gamma (IFN-γ) canonical signaling pathway. Upon ligand binding, IFNγR1 and IFNγR2 oligomerize and transphosphorylate, activating Janus activated kinase (JAK) 1 and JAK2. These, in turn, phosphorylate IFNγR1, creating a docking site for the signal transducer and activator of transcription (STAT) 1. Phosphorylated STAT1 homodimerizes in an antiparallel configuration, forming a complex gamma-activated factor (GAF), which translocates to the nucleus and binds to gamma-activated site (GAS), located at the promoters of primary response genes, increasing their transcription. Upon induction, transcription factor interferon-regulatory factor 1 (IRF1) binds to interferon-stimulated response element (ISRE) and enhances the transcription of several secondary response genes responsible for several immunomodulatory functions. Suppressor of cytokine signaling (SOCS) proteins negatively regulate the IFN-γ pathway by inhibiting JAKs and STAT1 phosphorylation. Through dephosphorylation and deacetylation, the configuration of STAT1 homodimers reverts to parallel, triggering their exit from the nucleus.

The IFN-γ receptor is composed of two ligand-binding IFNγR1 chains associated with two signal-transducing IFNγR2 chains, which are responsible for connecting to the cytoplasmic transduction machinery (see Figure 1). The *IFNGR1* and *IFNGR2* are localized in chromosome 6 and 21, respectively, and their expression differs significantly. While IFNγR1 is constitutively expressed at moderate levels on the surface of almost all cells, IFNγR2 is constitutively expressed at low levels, and its expression is tightly regulated, according to the state of cellular differentiation or activation (66). For example, CD4 T helper cell subsets differ in their ability to respond to IFN-γ (67, 68). Remarkably, IFN-γ activates the signal transducer and activator of transcription (STAT) 1 that maintains the expression of T-bet, the master transcription factor that controls IFN-γ expression in T cells (69). This signaling constitutes a positive feedback loop that maximizes Th1 immunity (70–72). Notably, Th1 cells are more resistant to the antiproliferative effects of IFN-γ than Th2 cells. This is likely due to lower levels of expression of the IFNγR2 subunit that allows Th1 cells to continue to proliferate during IFN-γ signaling. By contrast, Th2 cells that do not produce IFN-γ express higher levels of the IFNγR2 subunit, rendering them particularly susceptible to the presence of IFN-γ that inhibits their proliferation (67, 68, 73). Nevertheless, IFNγR2 downregulation may be also induced in Th2 cells when they are exposed to IFN-γ (68). Thus, IFN-γ appears to regulate the expression of its own receptor on specific cell types, representing a regulatory mechanism of cellular desensitization in response to cytokines present at the local microenvironment. As a result, IFNγR2 expression can be a limiting factor in IFN-γ responsiveness and functional outcome that can dictate the Th1–Th2 phenotype switch and modulate the subsequent immune response.

### JAK/STAT Signaling Pathway

The biological effects of IFN-γ are elicited through activation of intracellular molecular signaling networks, mainly *via* the JAK/STAT pathway, which modulates the transcription of hundreds of genes and mediates diverse biological responses (50, 74–76). Upon IFN-γ binding, the intracellular domains of IFNγR2 oligomerize and transphosphorylate with IFNγR1, activating the downstream signaling components, JAK1 and JAK2. The activated JAKs phosphorylate the intracellular domain of the receptor (tyrosine 440 on human IFNγR1), creating binding sites for STAT1 (77). STAT1 is then phosphorylated in the C-terminus on tyrosine Y701 residues by JAK, resulting in the formation of STAT1 homodimers complexes, known as gamma-activated factors (GAFs), which translocate to the nucleus and regulate gene expression through binding to gamma-activated site (GAS) elements in the promoters of interferon-stimulated genes (ISGs) (78). One of the major primary response genes induced by STAT1 signaling is the transcription factor interferon-regulatory factor 1 (IRF1), a member of the IFN regulatory transcription factor family (79). IRF1 functions as a transcription activator of interferon-stimulated response elements (ISRE), leading to the transcription of a large number of secondary response genes (Figure 1). For instance in breast cancer cells, a genome-wide identification of IFN-γ-induced IRF1 activation reveals over 17,000 binding sites, with “apoptosis” or “cell death” as the most enriched target processes underlying the direct tumoricidal property of the cytokine (80). However, tumor cells also develop resistance to IFN-γ through differential IRF1 responsiveness, pointing out that the JAK/STAT signaling pathway needs to be tightly regulated to avoid detrimental consequences of excessive stimulation and highlighting its role on immune responses and tumorigenesis (81). STAT1 targets of the IFN-γ-mediated signaling also include the SMAD family member 7 (SMAD7), and proteins involved in cell cycle regulation, such as c-Myc and the cyclin-dependent kinase inhibitor 1A (82–84).

The JAK/STAT signaling pathway is regulated at several levels by positive and negative mechanisms. In particular, deregulation or inhibition of the JAK/STAT pathway leads to lowered immunity and is often associated with increased tumorigenesis (85, 86) or metastatic dissemination (87). STATs are also involved in the development and function of the immune system and play a role in maintaining tumor surveillance [reviewed in Ref. (88)]. STAT1, as a tumor suppressor, is deducted for its expression in tumor cells, modulates their immunological status and consequently their response to antitumor immune responses. Indeed, STAT1-deficient tumor cells were more susceptible to NK cells while STAT1-proficient tumor cells were more sensitive to CD8^+^ T cells (89). In the same way, STAT1-deficient mice that are impaired in Th1 cell polarization, exhibited reduced IFN-γ expression and compromised cytolytic and NK lytic activity, failing to control tumor growth in contrast with wild-type mice (90). In addition, cell-autonomous tumor-suppressor functions of STAT1 have also been reported in breast cancer (91). However, there is growing evidence that STAT1 also acts as a tumor promoter (92–94) since it can enhance resistance to chemotherapeutic agents and radiation in carcinoma (95). Importantly, STAT1 also participates in the signaling from different cytokines, including IL-21, IL-27, and IL-35. These cytokines have been proposed to limit antitumor immunity in specific cellular, molecular, and microenvironmental contexts (96–101). Thus, STAT1 phosphorylation reflects not only the threshold and magnitude of IFN-γ response but also of other immune mediators, highlighting the importance of the regulation of STAT1 phosphorylation. One of the most important negative regulators of the JAK/STAT signaling pathway is the suppressor of cytokine signaling (SOCS) proteins, which expression is increased in response to IFN-γ signaling through IRF1 (102, 103). SOCS blocks the activity of JAKs by a negative feedback loop, but also regulates other cytokines downstream signaling. SH2 domains in SOCS proteins directly bind to phosphorylated tyrosine residues of activated JAKs, blocking the recruitment of signal transducer adaptors, such as STATs, and JAK activity (102). Furthermore, SOCS promote interactions that lead to ubiquitination and proteasome degradation of components of the JAK/STAT signaling (104, 105). SOCS1 even prevents regulatory T (Treg) cells from producing IFN-γ by suppression of STAT1, avoiding the conversion of Treg cells into effector cells (106). In addition, SOCS2-deficient mice showed a reduction in lung metastases and an increase in survival following melanoma challenge (107).

Alternatively, the transcriptional activity of STAT1 can be positively regulated by other signaling cascades triggered by IFN-γ binding, such as the mitogen-activated protein kinase pathway, protein kinase C, and PI3K/AKT, which phosphorylate STAT1 in its transactivation domain (108). Adding to the complexity, under certain circumstances, IFN-γ also can activate STAT1-independent pathways through other transcription factors, namely STAT3 (109), STAT5 (110), nuclear factor-kappa B (NF-κB) (111), and activator protein 1 (112). In conclusion, the primary response of IFN-γ is mediated by GAF that acts on genes with GAS binding sequence in their promoter, while the primary response of type I IFNs is mediated by ISGF3 (STAT1/STAT2/IRF9 complex) that induces genes that have ISRE in their promoter. Thus, some of the ISGs are regulated by both types of IFNs, whereas others are selectively regulated by each type of IFN, consequently potentiating the diversity of biological responses.

## Biological Functions

### IFN-γ Actions on Immune Cells

**Figure 2:**
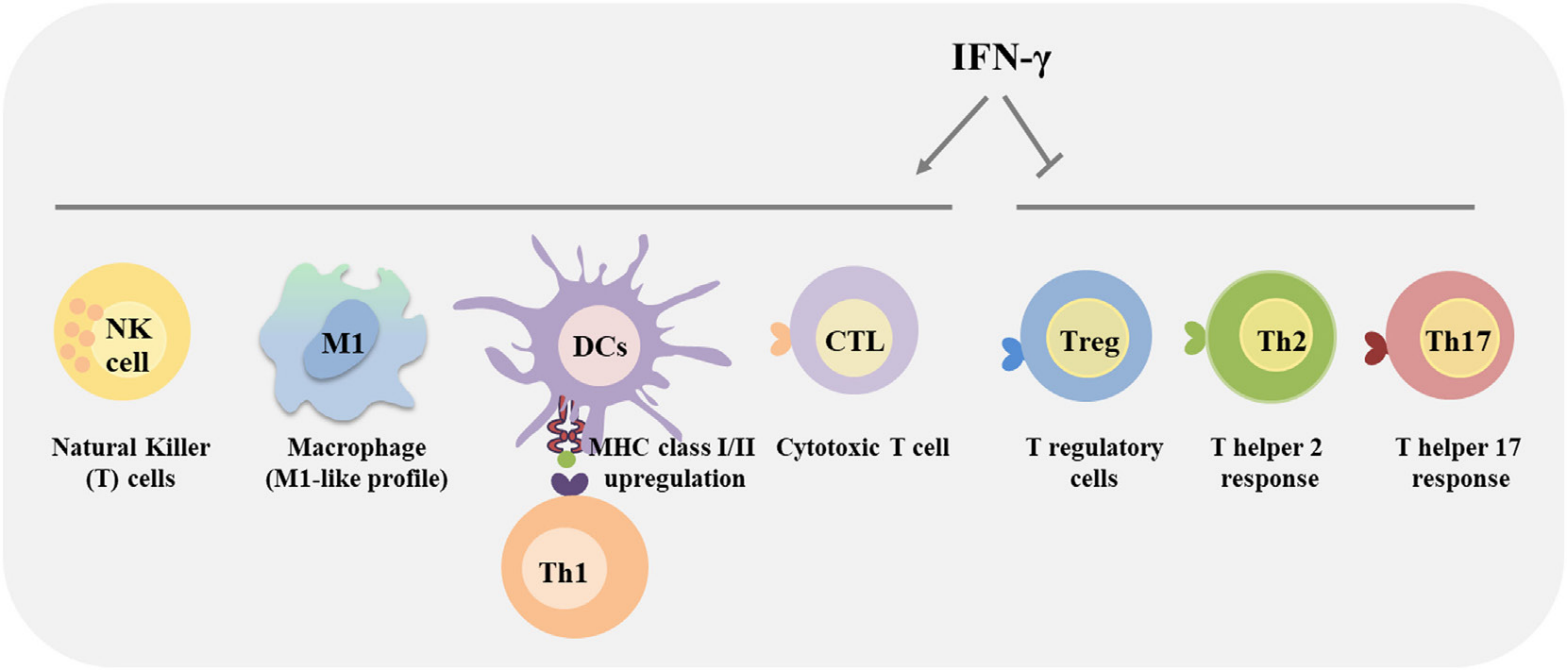
Immunomodulatory effects of interferon-gamma (IFN-γ). IFN-γ produced by immune cells affects the behavior of distinct immune cells within the tumor microenvironment. Specifically, IFN-γ plays a major role in activating anticancer immunity, by promoting the activity of CD4 T helper type 1 cells, CD8 cytotoxic T lymphocyte (CTL), natural killer (NK) cells, dendritic cells (DCs), and macrophages, promoting the antigen presentation. Additionally, IFN-γ activates macrophages towards a more pro-inflammatory and tumoricidal phenotype (M1-like). Alternatively, IFN-γ inhibits regulatory T (Treg) cells, Th2 and Th17 differentiation and functions.

Interferon-gamma signaling pathway coordinates several biological responses, primarily involved in host defense and immune surveillance but also in the establishment of adaptive immunity (Figure 2) and in the regulation of inflammation, apoptosis and cell cycle. One of the first described biological effects of IFNs was the upregulation of the major histocompatibility complex (MHC) molecules (113, 114) as well as the upregulation of the whole MHC I and II antigen processing and presentation machinery including transporter associated with antigen processing (TAP) 1/2, invariant chain, and the expression and activity of the proteasome (115–122). Furthermore, in some tumor types, such as multiple myeloma and melanoma cells, IFN-γ can also upregulate the MHC class II transactivator (CIITA) that leads to MHC class II expression (123, 124). Thus, IFN-γ initiates an immune-antigenic exposure program in the target cells, and this ensures the rapid recognition of stressed tissues. IFN-γ is a major product of Th1-mediated immune response and orchestrates Th1 effector mechanisms, as further activation of innate immunity (macrophages and NK cells) in a positive feedback loop. Upregulation of cell surface MHC class I by IFN-γ is crucial for host response to intracellular pathogens and tumor cells, due to cytotoxic T cell activation, promoting cell-mediated immunity. IFN-γ directly acts as a cytotoxic CD8 T cell differentiation signal, and it is essential for the induction of cytotoxic T cell precursor proliferation (125, 126). IFN-γ also upregulates cell surface MHC class II on APCs, thus promoting peptide-specific activation of CD4 T cells (25, 127–129). In addition, IFN-γ activates macrophages toward a pro-inflammatory profile, exhibiting an increased phagocytic ability as well as enhanced microbial killing activity (130). In fact, IFN-γ was initially shown to induce “classical” activation of macrophages and polarization toward a tumoricidal phenotype (131). Interestingly, the original name of IFN-γ was macrophage activation factor (132, 133). IFN-γ controls specific gene expression programs involving more than 290 genes related to cytokine and chemokine receptors, cell activation markers, cellular adhesion proteins, MHC proteins, proteasome formation, protein turnover, and signaling mediators and regulators (134). The ability of IFN-γ to induce tumor cell killing includes the activation of the NADPH-dependent phagocyte oxidase system, nitric oxide production, tryptophan depletion and upregulation of lysosomal enzymes (121, 135, 136). These events result in recruitment of effector cells to help in the inflammation resolution process (137, 138). In addition, as a major cytokine of Th1 cells, IFN-γ maintains Th1 lineage commitment through a positive feedback loop that stabilizes the Th cell phenotype (72, 139–141) and cross-inhibits the differentiation to other Th cell subsets (Figure 2). Indeed, IFN-γ inhibits Th2 cell differentiation (142, 143) and consequently IL-4 production. This regulation involves the inhibition of the IL-4/STAT6 pathway, required for Th2 cell differentiation, and it is mediated at least by IFN-γ-induced SOCS1 that inhibits IL-4R signaling (144, 145). Furthermore, IFN-γ-induced T-bet inhibits Th2 cell differentiation by directly interfering with the activity of Th2 cell-specific transcription factor, GATA-3 (146). Höfer and colleagues, using mathematical models, proposed that IL-4 also acts to propagate Th2 cell differentiation (147). A high IL-4 level promotes increased GATA-3 expression that further enhances GATA-3 transcriptional imprinting for Th2 differentiation (147, 148). This model proposed that high expression state of GATA-3 can be suppressed by strong inhibition of autoactivation, as observed in the presence of Th1-polarizing conditions (147, 149). IFN-γ was also described to downregulate the IL-4-inducible gene expression (150). The cross-regulation of Th1 and Th2 cells was also demonstrated in STAT6-deficient mice, which lack Th2 phenotype and associated immune responses. These animals displayed augmented tumor-specific IFN-γ production and cytotoxic T cell activity and, consequently rejected the tumor cell line that grew progressively in the wild-type control (151).

Interferon-gamma produced by Th1 cells also counteracts Th17 cell development and their effector functions (152–154). Several mechanisms can be considered as the inhibition of molecules involved in the Th17 differentiation (155, 156), the inhibition of STAT3 by STAT1 (157) and recently, T-bet was demonstrated to prevent differentiation of Th precursors into Th17 cells by blocking the expression of the Th17 cell lineage-specific transcription factor, RORγt (158). Furthermore, IFN-γ also exerts regulatory functions to limit tissue damage associated with inflammation (63, 159–162) (Figure 2). IFN-γ has been classically considered as a pro-inflammatory cytokine, involved in the regulation of anti-inflammatory responses, by antagonizing the IL-10 (157, 163) and TGF-beta (164) signaling pathways. Consequently, IFN-γ inhibits Treg cell differentiation and functions (165, 166). However, in some chronic inflammation conditions, IFN-γ plays a crucial role in attenuating tissue destruction. In this case, IFN-γ might be protective (62, 167) by promoting the number and function of Treg cells (168–170). In addition, IFN-γ production by Treg cells themselves was shown to be a key feature of the Treg cells that are capable of dampening Th1 cell responses (171–174). Thus, IFN-γ dictates the differentiation of specialized Foxp3^+^T-bet^+^ Treg cells that selectively suppress Th1 cells, and constitute a negative feedback loop to minimize the detrimental effect of IFN-γ. IFN-γ also promotes the differentiation of myeloid-derived suppressor cells (MDSCs) that restrain overactivation of effector T cells, maintaining tissue homeostasis (175, 176). Other regulatory mechanisms involving IFN-γ signaling that dampen the magnitude of the immune response have been reported, as the induction of indoleamine 2,3-dioxygenase (IDO) by Treg cells, monocytes and stromal cells (177–180), and of the programmed cell death 1 (PD-1) ligand (PD-L1) on immune and transformed cells, inhibiting T cell responses (181–183).

### IFN-γ Actions on Transformed Cells and on the Tumor Microenvironment

Interferon-gamma is involved in antiproliferative (18), antiangiogenic (184) and pro-apoptotic effects established against neoplastic cells. How IFN-γ induces the signaling pathways initiating and propagating the apoptotic cascade remains to be elucidated. The level of complexity is demonstrated by the fact that the mechanism might depend on the tumor cells themselves. For example, while in a glioblastoma cell line the induction of apoptosis was due to suppression of the PI3K/AKT pathway, in another glioblastoma cell line apoptosis occurred independently of the PI3K/AKT pathway but required NF-κB (185). It was also shown that IFN-γ induces apoptosis of human pancreatic carcinoma cells in a caspase-1-dependent manner (186). A review covered in detail the mechanism of induction of programmed cell death (187). So far, the known biological functions of IFN-γ indicate that, although it can act as a potent inducer of antitumor immunity, it actually has a dual role and may also favor tumor immune evasion.

## IFN-γ in Cancer

The first reports pointing to the relevance of IFN-γ in antitumor immunity came from studies with the fibrosarcoma (Meth A) cell line, refractory to IFN-γ signaling, since it lacks the expression of the IFNγR1 subunit. IFN-γ-insensitive Meth A cells displayed enhanced tumorigenicity compared with control cells and were not rejected in syngeneic tumor mice models, suggesting that IFN-γ plays an important role in tumor cell elimination (18). This finding was further supported by experiments using 129/SV IFN-γ insensitive mice, lacking the IFNγR1 subunit or STAT1, which developed 3-methylcholanthrene (MCA)-induced sarcomas more rapidly and more frequently than their wild-type counterparts (12). Similarly, these IFN-γ-insensitive mice lacking the tumor-suppressor protein p53 formed spontaneous tumors more rapidly than IFN-γ-sensitive p53-deficient mouse (12). In addition, C57BL/6 mice that lack the gene encoding IFN-γ also displayed higher susceptibility to experimental (B6, RM-1 prostate carcinoma) and spontaneous (BALB/c, DA3 mammary carcinoma) models of primary and metastatic tumors (13, 14). Notably, further studies described that IFN-γ may cooperate with other molecules to prevent tumor formation. Mice deficient in both granulocyte/macrophage colony-stimulating factor (GM-CSF) and IFN-γ developed lymphoma and non-lymphoid solid tumors at a higher rate than did mice deficient in GM-CSF or IFN-γ alone (15). Additional studies revealed that mice insensitive to IFN-γ, or that lack the recombination activating gene (RAG) protein (failing to produce mature B and T lymphocytes), or that lack both, showed similar incidence of MCA-induced sarcomas, suggesting that the T cell–IFN-γ axis is involved in immune surveillance (10).

The role of IFN-γ on cancer immunoediting emerged from studies assessing the immunogenicity of tumors from immunocompetent versus immunodeficient mice. Kaplan *et al*. showed that MCA-induced sarcoma cells from IFNγR1*-*deficient mice (unresponsive to IFN-γ signaling) grow as aggressively in immunocompetent as in IFNγR1-deficient mice. However, when IFN-γ responsiveness was conferred on the tumor cells by introducing the IFNγR1 subunit, they became more immunogenic and were rejected through a T cell-dependent manner (12). This constitutes the first demonstration that IFN-γ sensitivity of the tumor is fundamental for an efficient antitumor response. Other studies revealed that wild-type hosts rejected 40% of MCA-induced sarcomas derived from RAG2-deficient mice, showing that these tumors were more immunogenic than those from wild-type mice (10). In addition, human tumors were evaluated for their ability to upregulate MHC I expression in response to IFN-γ stimulation. These studies revealed that 33% of 33 melanoma tumor cell lines showed a reduction in IFN-γ sensitivity while 4 of 17 lung adenocarcinoma cell lines were totally unresponsive to IFN-γ (12). This lack of response resulted from cellular defects on IFNγR1 and of JAK proteins and may explain the ability of many tumor cells to evade the immune response. Recently, JAK1/2 deficiency was demonstrated to protect melanoma cells from antitumor IFN-γ activity and results in T-cell-resistant melanoma lesions (188). Others reported the lack of STAT1 in melanoma cell lines and in some chronic myeloid leukemia cells (189). Furthermore, DNA methylation that selectively represses CIITA, in colorectal and gastric cancer cell lines, was associated with the absence of IFN-γ-induced HLA-DR, suggesting that this epigenetic alteration of CIITA enables some gastrointestinal cancer cells to evade the immune system (190). Concomitantly, epigenetic alterations repressing *MHC2TA* were described in T cell leukemias, B cell lymphomas, and in several cancer cells, such as small cell lung cancer and neuroblastoma cells that were unable to express MHC II upon IFN-γ stimulation (191–194). Consistently, IFN-γ upregulates CIITA expression on multiple myeloma and melanoma cells increasing their MHC II expression (123, 124). These findings indicate that IFN-γ acts on tumor cells, enhancing their recognition by CD8 T cells as well as by CD4 T cells, and unveiling a key role in the promotion of tumor immunogenicity. Altogether, these works pave the way for the elaboration of the stepping-stone concept of immunoediting promoted by IFN-γ (195, 196).

### IFN-γ-Mediated Mechanisms Underlying Antitumorigenic Effects

As described earlier, the mechanisms by which IFN-γ exerts its antitumor effects depend on multiple processes. IFN-γ is described as an antiproliferative agent that regulates the expression of cyclin-dependent kinase inhibitor 1 (p21) through STAT1 activation in tumor cells (84, 197). Moreover, IFN-γ is able to promote tumor cells apoptosis by upregulating the expression of caspase-1, -3, -8 (198, 199) and by enhancing the secretion of FAS and FAS ligand (200) and TNF-related apoptosis-inducing ligand (201, 202). Recent studies showed that IFN-γ also induces its tumoricidal effects through a form of regulated necrotic death (also named as necroptosis) that relies on the activity of the serine–threonine kinase RIP1 (203). Importantly, IFN-γ is also involved in the inhibition of angiogenesis, impairing the proliferation and survival of endothelial cells, inducing ischemia in the tumor stroma (184, 204, 205). In particular, IFNγR is expressed on blood endothelial cells and engagement of the receptor results in blood vessel destruction and necrosis, an important mechanism that leads to tumor rejection (206).

Considering the effect of IFN-γ on the host immune cells present at the tumor microenvironment, major efforts have been made for the development and establishment of combined clinical therapeutic applications (90, 151, 207). IFN-γ is critical for T cell, NK and NKT cell trafficking into the tumors through CXCL9, CXCL10, and CXCL11 chemokine induction (208, 209). Accordingly, T cells fail to migrate to tumor site in IFNγ-deficient mice (65). In commitment, dipeptidylpeptidase 4 inhibition, a protease that inactivates these chemokines, enhanced tumor rejection by increasing lymphocytes trafficking into the tumor (210). Lately, galectin-3 secreted by several tumors was demonstrated to bind glycosylated IFN-γ at the tumor extracellular matrix, avoiding IFN-γ diffusion and the formation of an IFN-γ-induced chemokine gradient required for T cell recruitment and infiltration (211). In addition, CXCL10 also prevents tumor angiogenesis by blocking endothelial cell proliferation (212) and consequently a decrease in microvessel density as observed in melanoma tumor xenografts (213). Apoptosis of endothelial cells by IFNs causes restriction of blood flow within the tumor vasculature, leading to tumor shrinkage (214). This is an effect of IFN-γ, not directly targeted to the tumor cell, but to the tumor vasculature, with drastic and desirable effects on tumor growth. A recent report also showed that IFN-γ was essential for the initial priming and differentiation of cytotoxic T cells residing in the periphery of the eye, contributing to the regression of intraocular tumors (215). Supporting data from therapy models showed that IFN-γ induces *survivin* and *ifi202*, two genes involved in T cell maturation, survival, and proliferation, in tumor-specific T cells (216). Overall, these studies demonstrated the relevance of IFN-γ on T cell-mediated antitumor immunity.

Interferon-gamma is also involved in macrophages tumoricidal activity (217). This cytokine supports a CD4 T cell/macrophage effector axis which acts as immune surveillance mechanism for MHC II-negative cancer cells (25). Indeed, upon recognition of tumor antigens present in the context of MHC II by macrophages, CD4 T cells secrete IFN-γ that further activates macrophages in the tumor, leading to tumor growth inhibition (25). This collaboration between CD4 T cells and macrophages was also essential for successful cancer immune surveillance in non-solid cancers, as myeloma and B-cell lymphoma. Indeed, Th1-secreted IFN-γ was shown to trigger a cytotoxic activity of tumor-associated macrophages (TAMs) and also induces CXCL9/MIG and CXCL10/IP-10 secretion by macrophages, which may affect the tumor progression by angiogenesis inhibition (129). IFN-γ-activated macrophages also acquire a tumoricidal phenotype with the upregulation of cytotoxicity-associated markers including granzyme A/B, and NKG2D (129). In addition, in STAT6-deficient mice, that display increased levels of IFN-γ, rejection of metastatic disease after removal of the primary tumor involved the generation of pro-inflammatory macrophages, also termed M1-like macrophages, and a decrease in MSDCs that accumulated during primary tumor formation (218). Studies from APC^Min/+^ mice (that are highly susceptible to spontaneous intestinal adenoma formation) lacking IFN-γ signaling showed an accumulation of TAMs, more prone towards protumoral (M2-like) polarization, and upregulation of matrix metalloproteases. These results suggest that IFN-γ unresponsiveness contributes to the creation of an anti-inflammatory microenvironment, favorable to intestinal tumorigenesis (219). The properties of IFN-γ to reverse the myeloid immunosuppressive functions were also demonstrated in protumor role of human ovarian TAMs (220) and human M2-like macrophages (221).

Importantly, IFN-γ has also a key role on IL-12 production, supporting the activity of this later cytokine in cancer immune surveillance (222–225). Indeed, exogenous IL-12 administration into fibrosarcoma-bearing mice resulted in a complete tumor regression (222). This observation was extended to primary tumorigenesis models treated with exogenous IL-12 (226, 227). Consistent with this, chimeric antigen receptor-redirected T cells engineered to produce IL-12 where found to secrete increased IFN-γ levels and to display enhanced antitumor cell activity (228–230).

Regarding the importance of IFN-γ in cancer diagnostics, IFN-γ-associated signatures have a predictive value in cancer immune phenotypes (81, 231, 232). In addition, IFN-related gene signature is a predictive marker for chemotherapy and radiotherapy efficiency for breast cancer (94) as well as to PD-1 or cytotoxic T lymphocyte antigen-4 (CTLA-4) blockade in various types of malignancies (233–235). Consistently, immunotherapy using immune checkpoint blockers (anti-CTLA-4 and/or anti-PD-1) combined with anticancer vaccines, clearly associate inhibition of tumor growth with increased proportion of IFN-γ-producing effector T cells (236, 237). This is also verified in clinical trials, through which the anti-CTLA-4 therapy was associated with an increase of IFN-γ-producing ICOS^+^ (inducible costimulatory) CD4 T cells and of T effector/Treg cell ratio in bladder cancer samples (238). In addition, PD-1 blockade was demonstrated to enhance T cell infiltration by promoting IFN-γ-inducible chemokines (239). In other way, it was recently shown that IFN-γ-induced Treg cell fragility (loss of suppressive function) is required for response to anti-PD-1 therapy (240).

Altogether, the versatility of IFN-γ and its fine-tuned biological effects highlight its relevance for therapeutic applications, and some clinical trials have already encouraging results. In fact, 75% of metastatic melanoma patients were non-responders to anti-CTLA-4 therapy, and this was associated with genomic defects of IFN-γ signaling genes on tumors (241). Recently, apelin receptor (*APLNR*) was described to regulate JAK/STAT signaling, modulating IFN-γ responses. Multiple loss-of-function mutations in *APLNR* were identified in patient tumors refractory to immunotherapy (242). The inclusion of IFN-γ in the first-line treatment of ovarian cancer resulted in benefit regarding progression-free survival, with acceptable toxicity (243). IFN-γ treatment also appears to be effective against bladder tumors by recruitment and activation of intratumoral leukocytes (244). In a phase I clinical trial, which combined adoptive T cell therapy with intralesional administration of adenovirus expressing IFN-γ in metastatic melanoma, 38.5% of the patients had an overall objective response and 46% were able to control the disease (245).

### IFN-γ-Mediated Mechanisms Underlying Protumorigenic Effects

It is becoming increasingly clear that IFN-γ can exert certain effects supporting tumorigenesis. Immune evasion can operate through tumor cells losing responsive to IFN-γ signaling to avoid its antiproliferative, pro-apoptotic, and immunoregulatory actions. This has been demonstrated with the tumor cells losing the receptor for IFN-γ or a component of JAK/STAT signaling (12, 18). In addition, constitutive activation of inhibitory molecules of this pathway, as SOCS1 and SOCS3, limits the actions of IFNs on human melanoma cells (246) and favors the activation of alternative signaling pathways, as STAT3, which is associated with tumor progression (247). These evidences suggest that tumor cells develop IFN-γ-dependent strategies to evade the immune system, leading to the emergence of very aggressive tumors, which are on the basis of immunoediting. In 2011, Zaidi and Merlino proposed that IFN-γ actions might play a physiological role in protecting cells from damage in a setting of tissue remodeling and repair, while on cells harboring oncogenic mutations, the same mechanisms may prevent cell destruction and allow complete transformation (248). Consistent with this, NF-κB in tumor cells was shown to act as a protective mechanism against IFN-γ-induced necroptosis (203).

Indeed, there are significant evidences that tumor cells can take the advantage of IFN-γ as an inducer of anti-inflammatory responses and protumor effects. The first report of the negative potential effects was in 1987 by Taniguchi and colleagues who proposed that IFN-γ changes the metastatic ability of the B16 melanoma cells in a cell-autonomous manner (249). Data from experiments using the CT26 colon carcinoma model showed that IFN-γ promotes tumor escape through the downregulation of the endogenous tumor antigen gp70 (250). IFN-γ expression by human melanoma samples was associated with enhanced expression of MHC class II molecules and the acquisition of a more aggressive phenotype (251, 252).

One of the principal mechanisms of tumor immune escape is the suppression of cytotoxic T cells and of NK cell-mediated immune responses. Brody and colleagues showed that IFN-γ upregulates IDO in melanoma cells and recruits Treg cells to avoid immune recognition (253). Curiously, IFN-γ induced IDO competence on human monocyte-derived DCs but had no effect on pro-inflammatory cytokine release, suggesting that IFN-γ triggers IDO activity and pro-inflammatory cytokine release as distinct cellular programs. In addition, IDO-competent DCs induced regulatory activity on allogeneic T cells (179). IFN-γ was also described to be involved in the accumulation of MDSCs in inflamed liver, which leads to T cell suppression (254). MDSCs producing nitric oxide decreased IFN-γ responsiveness of immune cells, such as T and NK cells (255).

One important aspect is the ability of IFN-γ to induce PD-L1 expression in cancer, stromal and myeloid cells to impair effector tumor immunity (181). Abiko and colleagues demonstrated that the contact between tumor cells and CD8 T cells is necessary for the induction of PD-L1, underlying the importance of paracrine exposure to IFN-γ (256). Recent reports suggest that loss of IFN-γ pathway genes, such as JAK1 and JAK2, is associated with resistance to anti-PD-1 therapy (257, 258). Prolonged IFN-γ signaling in tumors was also shown to coordinate PD-L1-dependent and PD-L1-independent resistance to immune checkpoint blockade and to other therapeutic combinations, such as radiation and anti-CTLA-4, through a multigenic resistance program (259). In addition, other inhibitory pathways are reinforced by IFN-γ, including CTLA-4 and CD86/CD80 interaction (260).

Interferon-gamma was used in clinical trials for melanoma but no significant improvement for patients was observed (261–264). In fact, IFN-γ treatment had no contribution to the outcomes of patients with metastatic renal cell carcinomas (265), leukemia (266), pancreatic carcinoma (267), breast cancer (268), or into the postoperative surgical therapy for colon cancer (269). Furthermore, a phase 3 trial of IFN-γ plus standard treatment with carboplatin/paclitaxel versus carboplatin or paclitaxel alone, for treated advanced ovarian tumors, was early terminated due to a higher incidence of serious hematological toxicities in patients receiving combined therapy compared with chemotherapy alone (270). The failed attempts to treat cancer patients with exogenous IFN-γ raised several concerns: the absence of tumor immunogenicity, the lack of IFN-γ-signaling components, the upregulation of IFN-γ signaling inhibitors, the immunosuppressive tumor microenvironment, the lack of effector T cells, or presence of anergic T cells and, in some cases toxicity. These accumulating evidences reinforce the importance to determine the grade of patients’ IFN-γ-responsiveness. For example, in cases with low IFN-γ actions, active immunization either *via* IFN-γ treatment or *via* adjuvants of the immune system, as toll-like receptor ligands, should be considered, as demonstrated recently by using bacterial outer membrane vesicles that eradicate established tumors in an IFN-γ-dependent mechanism (271). The combination with radio- and chemotherapy is expected to be useful through immunogenic cell death that also elicits the innate immune system. Promising results were obtained with combination of low-dose 5-fluorouracil with recombinant interferon-gamma (IFN-γ) in patients with advanced hepatocellular carcinoma (272). In cases with high levels of IFN-γ signaling, the therapy with anti-PD-1/anti-PD-L1 is expected to be important.

**Figure 3:**
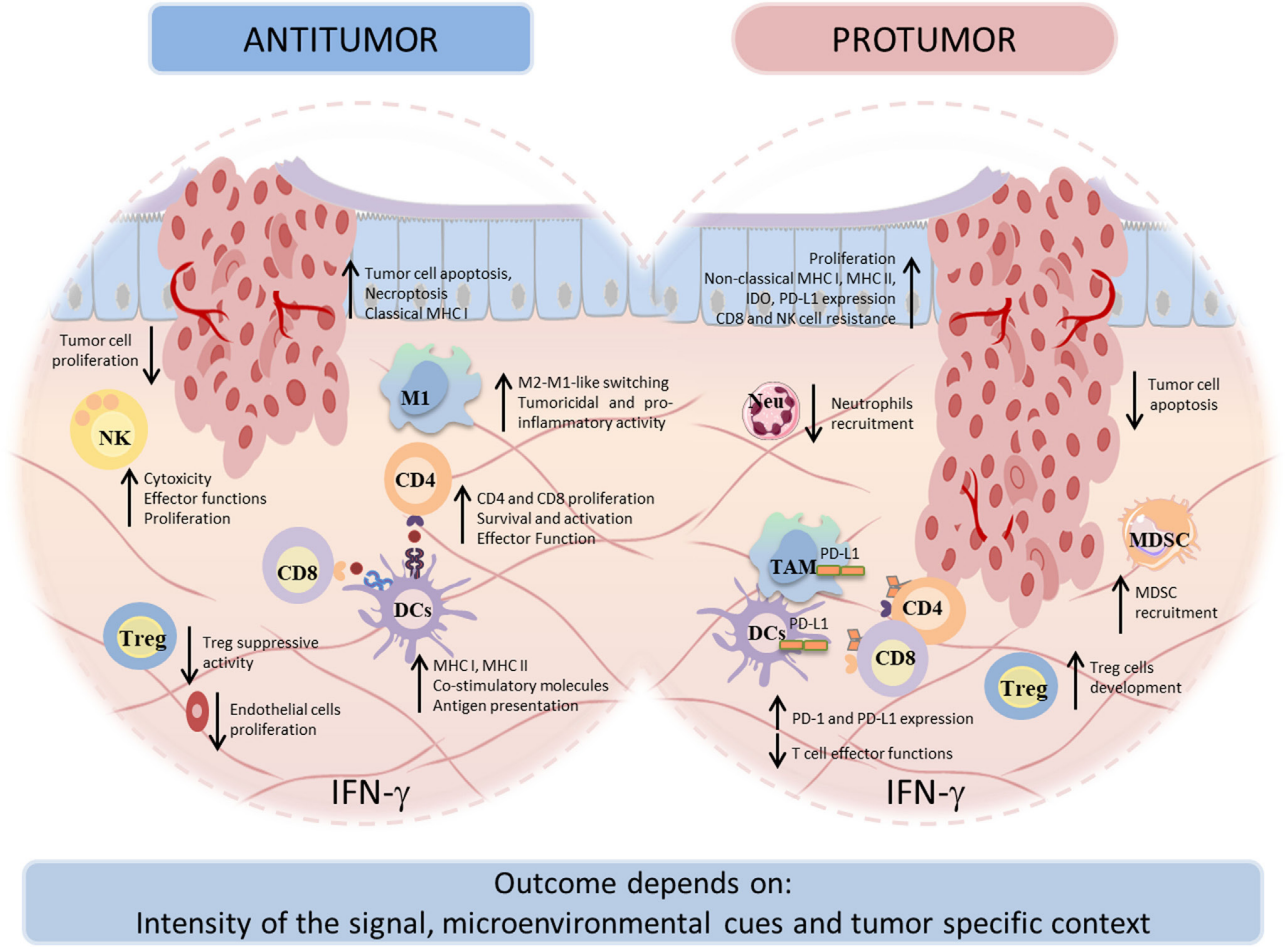
Dual face of interferon-gamma (IFN-γ) in tumor immunity. IFN-γ can display both antitumor and protumor activities. Under both circumstances, IFN-γ influences tumor cells directly and indirectly, by activation of immune cells. The antitumor effects comprise the development, recruitment, and activation of innate immune cells as well as the activation and maintenance of effector T cells. The antitumor effects of IFN-γ result in direct inhibition of tumor proliferation, recognition, and elimination. In other way, the protumorigenic role of IFN-γ involves proliferative and antiapoptotic signals, as well as escape of the tumor cells from recognition and cytolysis by cytotoxic T lymphocytes (CTLs) and natural killer (NK) cells. The broad range of IFN-γ actions depends on the context of tumor specificity, IFN-γ-signaling intensity, and other microenvironment conditions.

Overall, these findings indicate that the local immune microenvironment of tumors is complex and variable and that for an effective therapy it is essential to evaluate, individually, the immune profile of patients or immune contexture [reviewed in Ref. (232, 273)], taking into account that it may evolve and modify throughout the anticancer therapy (Figure 3).

## IFN-γ in Therapy—Where are We and Where are We Going?

Interferon-gamma therapy has ensued in clinical applications approved by the Food and Drug Administration in the treatment of chronic granulomatous disease, in 1999 and severe malignant osteopetrosis, in 2000. Despite the promising therapeutic applications of IFN-γ in several settings, its limited success in cancer-immunotherapy trials might be due to cancer cell unresponsiveness to this cytokine, the failure to deliver it locally or with the adequate periodicity to achieve a therapeutic effect. Moreover, IFN-γ clinical use has also been restricted due to several limitations inherent to its molecular properties. Essentially, these include stability problems, such as acid degradation, and also the tendency to aggregate irreversibly under mild denaturing conditions, with subsequent loss of biological activity [the pharmacological aspect of IFN-γ is reviewed in Ref. (274, 275)]. Furthermore, IFN-γ is rapidly cleared from the blood when administered intravenously (276), requiring frequent re-administrations of high cytokine concentrations, to elicit an effective response at the target site, leading to systemic toxicity and side effects, such as fever, fatigue, nausea, vomiting, diarrhea, neurotoxicity, and leukopenia (277). These adverse effects are caused mainly by high serum concentration of the protein, due to an unequal distribution between body fluids and tissues (276) and, additionally, to the ubiquity of receptors which are expressed at the membrane of the majority of human cells (278, 279) and also to the existence of a circulating soluble form (which function remains elusive) (280).

**Figure 4:**
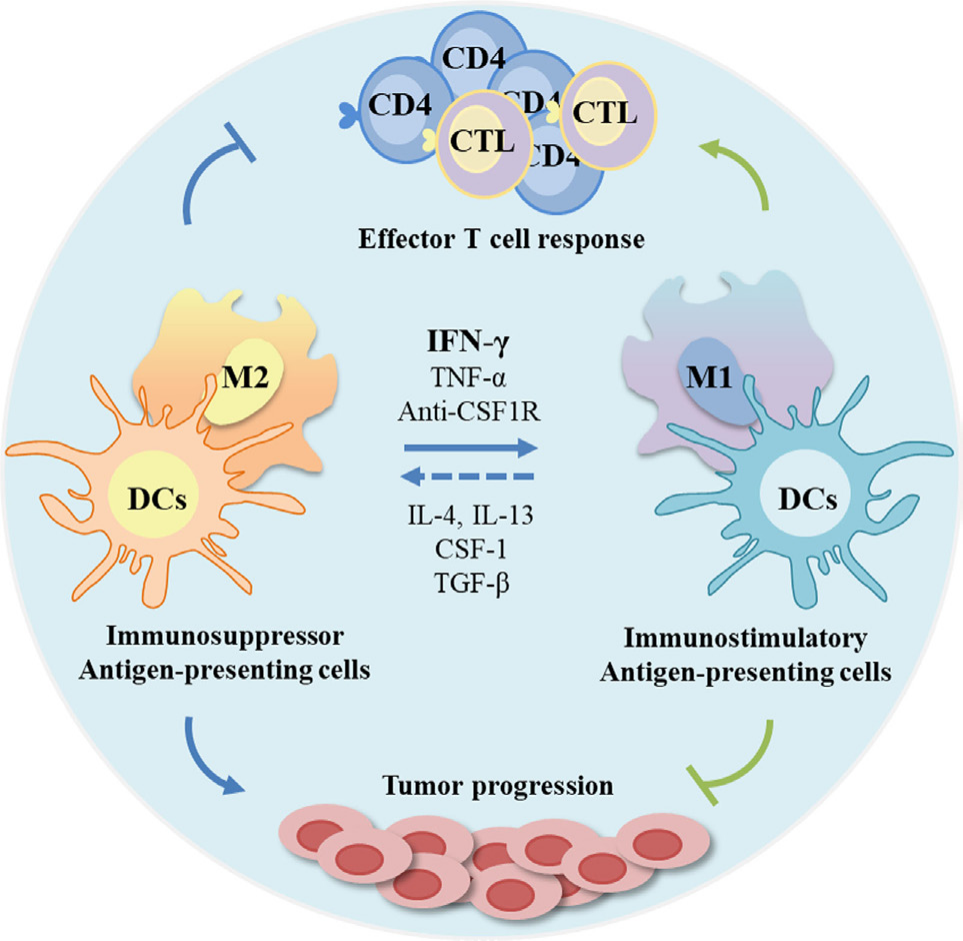
Modulation of antigen-presenting cells (APCs) profile as anticancer therapeutic strategies. The tumor microenvironment is frequently immunosuppressive with APCs functions compromised, and consequently with poor T cell response. As APCs can be modulated by microenvironmental signals, these cells are promising targets. Interferon-gamma (IFN-γ) and other molecules can be used to re-educate tumor-associated macrophages, frequently associated with anti-inflammatory status (M2-like) toward a pro-inflammatory and antitumor profile, while stimulating regulatory dendritic cells (DCs) to an immunostimulatory profile. This stimulation can potentiate effector T cell response and inhibit tumor progression.

These constraints in the clinical use of IFN-γ have encouraged the development of alternative delivery methods with the purpose of achieving higher therapeutic outcomes and, simultaneously, weaken its toxicity. Numerous reports have focused mainly on efficient routes of delivery rather than on systemic applications (281–287). In fact, IFN-γ is naturally produced in a paracrine manner, with local secretion and diffusion to the surrounding cells and microenvironment throughout the extracellular fluids (288). Therefore, a localized delivery of this cytokine has been determined to be more appropriated in terms of therapeutic efficiency, due to its specific effect at the target site, while simultaneously intensifying the intended cytotoxic effects and immunological stimulation (289). In particular, tumors can be rejected by local IFN-γ expression, but rejection of established tumors was less efficient over time, suggesting that timing of treatment plays a critical role, for transplanted tumors became less susceptible to local IFN-γ treatment the better they are established (206). Another relevant aspect concerns the mode of administration, being it an intermittent or sustained release. Several studies concluded that a sustained release strategy is more efficient by limiting the exposure of other cells and organs to the deleterious effects of high IFN-γ concentrations (290–295). In the particular case of cancer immunotherapy, consistent findings show that a stable and high concentration at the target site is required to elicit an effective response (288, 296), prompting several attempts to promote local delivery of IFN-γ with controlled release. These include liposomes, polymer gels, biodegradable microspheres, gene therapy, and magnetic or albumin nanoparticles (285, 297–301). However, these strategies revealed unsuccessful by failing to maintain a stable and/or bioactive cytokine prior release, an inadequate release rate, a labor intensive and cost ineffective manufacture, and safety issues. Oncolytic viruses have gained interest for immunotherapy due to their ability to selectively destroy tumor cells and to their potential to stimulate antitumor immunity. Oncolytic vesicular stomatitis virus expressing IFN-γ demonstrated greater activation of DCs, higher pro-inflammatory cytokines’ secretion, and reduced tumor growth in 4T1 tumor model compared with the parental virus, suggesting that specific production of the IFN-γ within the tumor microenvironment is beneficial for the antitumor immune response (302). Recently, an IFN-γ-delivery system based on chitosan/poly(γ-glutamic acid) polyelectrolyte complexes was described by our group to successfully decrease macrophage-derived stimulation of cancer cell invasion *in vitro* through the modulation of a pro-inflammatory macrophage phenotype (221). In fact, several efforts have been directed to educate APCs toward an immunostimulatory and antitumor phenotype (Figure 4) (303–306). In another work, a silk-based hydrogel was designed to regulate cytokine delivery for macrophages, which are actively involved in tissue remodeling and vascularization, with the aim to regulate the microenvironment of biomedical implants (307). Other potential strategy to improve the shorter half-live of IFN-γ is fusing it with antibodies, enhancing its stability in the serum and tumor target specificity and reducing toxic side effects (308). Although promising results have been achieved with some of these strategies, the desired requirements are yet to be accomplished and need further investigation/development.

## Concluding Remarks

Herein, we discussed the role of IFN-γ on tumor immunity and its potential therapeutic implications. On one side IFN-γ appears as a promoter of tumor immune surveillance and on the other as a supporter of tumor escape. The outcome of IFN-γ signaling depends on the tumor-specific context, the magnitude of the signal, and the microenvironmental cues. Nevertheless, IFN-γ or IFN-γ inducers remain promising agents to include in combined therapies against cancer. We believe that the effectiveness of future IFN-γ-based therapies will involve the development of systems to deliver the appropriate amount of cytokine to target cells, minimizing its side effects. In addition, these strategies would profit from the combination with conventional treatments and with anti-PD-L1 and anti-CTLA-4 therapies to overcome the regulatory effects of IFN-γ. Another important issue is to consider a personalized approach, which takes into account the patient responsiveness to IFN-γ, by using predictive biomarkers, as IFNγR2, SOCS, *APLNR*, STAT1, or STAT3. Thus, a comprehensive understanding of the complex and variable tumor microenvironment, as well as a deeper evaluation of the immune, vascular and stromal profile, will be necessary for the stratification of cancer patients and for the establishment of efficient personalized therapies.

## Author Contributions

FC performed the initial draft, written the manuscript, and designed Figures 2–4. AC written a part of Section “IFN-γ IN Therapy—Where Are We and Where Are We Going?” and performed Figure 1. RG, KS, and MO critically revised the manuscript, reorganized ideas, and approved the final version.

## Conflict of Interest Statement

The authors declare that the research was conducted in the absence of any commercial or financial relationships that could be construed as a potential conflict of interest.

## References

[1] Gresser I. Biologic effects of interferons. J Invest Dermatol (1990) 95:66S–71S. 10.1111/1523-1747.ep12874776 1701811

[2] Nagano Y Kojima Y [Immunizing property of vaccinia virus inactivated by ultraviolets rays]. C R Seances Soc Biol Fil (1954) 148:1700–2. 14364998

[3] Isaacs A Lindenmann J Virus interference. I. The interferon. Proc R Soc Lond B Biol Sci (1957) 147:258–67. 10.1098/rspb.1957.0048 26297790

[4] Ivashkiv LB Donlin LT. Regulation of type I interferon responses. Nat Rev Immunol (2014) 14:36–49. 10.1038/nri3581 24362405 PMC4084561

[5] de Weerd NA Samarajiwa SA Hertzog PJ Type I interferon receptors: biochemistry and biological functions. J Biol Chem (2007) 282:20053–7. 10.1074/jbc.R700006200 17502368

[6] Schroder K Hertzog PJ Ravasi T Hume DA. Interferon-gamma: an overview of signals, mechanisms and functions. J Leukoc Biol (2004) 75:163–89. 10.1189/jlb.0603252 14525967

[7] Saha B Jyothi Prasanna S Chandrasekar B Nandi D. Gene modulation and immunoregulatory roles of interferon gamma. Cytokine (2010) 50:1–14. 10.1016/j.cyto.2009.11.021 20036577

[8] Wack A Terczynska-Dyla E Hartmann R. Guarding the frontiers: the biology of type III interferons. Nat Immunol (2015) 16:802–9. 10.1038/ni.3212 26194286 PMC7096991

[9] Pott J Mahlakoiv T Mordstein M Duerr CU Michiels T Stockinger S IFN-lambda determines the intestinal epithelial antiviral host defense. Proc Natl Acad Sci U S A (2011) 108:7944–9. 10.1073/pnas.1100552108 21518880 PMC3093475

[10] Shankaran V Ikeda H Bruce AT White JM Swanson PE Old LJ IFNgamma and lymphocytes prevent primary tumour development and shape tumour immunogenicity. Nature (2001) 410:1107–11. 10.1038/35074122 11323675

[11] Wang L Wang Y Song Z Chu J Qu X. Deficiency of interferon-gamma or its receptor promotes colorectal cancer development. J Interferon Cytokine Res (2015) 35:273–80. 10.1089/jir.2014.0132 25383957

[12] Kaplan DH Shankaran V Dighe AS Stockert E Aguet M Old LJ Demonstration of an interferon gamma-dependent tumor surveillance system in immunocompetent mice. Proc Natl Acad Sci U S A (1998) 95:7556–61. 10.1073/pnas.95.13.7556 9636188 PMC22681

[13] Street SE Cretney E Smyth MJ. Perforin and interferon-gamma activities independently control tumor initiation, growth, and metastasis. Blood (2001) 97:192–7. 10.1182/blood.V97.1.192 11133760

[14] Street SE Trapani JA MacGregor D Smyth MJ. Suppression of lymphoma and epithelial malignancies effected by interferon gamma. J Exp Med (2002) 196:129–34. 10.1084/jem.20020063 12093877 PMC2194011

[15] Enzler T Gillessen S Manis JP Ferguson D Fleming J Alt FW Deficiencies of GM-CSF and interferon gamma link inflammation and cancer. J Exp Med (2003) 197:1213–9. 10.1084/jem.20021258 12732663 PMC2193978

[16] Mitra-Kaushik S Harding J Hess J Schreiber R Ratner L. Enhanced tumorigenesis in HTLV-1 tax-transgenic mice deficient in interferon-gamma. Blood (2004) 104:3305–11. 10.1182/blood-2004-01-0266 15292059

[17] Wheelock EF Interferon-like virus-inhibitor induced in human leukocytes by phytohemagglutinin. Science (1965) 149:310–1. 10.1126/science.149.3681.310 17533668

[18] Dighe AS Richards E Old LJ Schreiber RD. Enhanced in vivo growth and resistance to rejection of tumor cells expressing dominant negative IFN gamma receptors. Immunity (1994) 1:447–56. 10.1016/1074-7613(94)90087-6 7895156

[19] Kelker HC Le J Rubin BY Yip YK Nagler C Vilcek J. Three molecular weight forms of natural human interferon-gamma revealed by immunoprecipitation with monoclonal antibody. J Biol Chem (1984) 259:4301–4. 6423641

[20] Ealick SE Cook WJ Vijay-Kumar S Carson M Nagabhushan TL Trotta PP Three-dimensional structure of recombinant human interferon-gamma. Science (1991) 252:698–702. 10.1126/science.1902591 1902591

[21] Takaoka A Mitani Y Suemori H Sato M Yokochi T Noguchi S Cross talk between interferon-gamma and -alpha/beta signaling components in caveolar membrane domains. Science (2000) 288:2357–60. 10.1126/science.288.5475.2357 10875919

[22] Mitani Y Takaoka A Kim SH Kato Y Yokochi T Tanaka N Cross talk of the interferon-alpha/beta signalling complex with gp130 for effective interleukin-6 signalling. Genes Cells (2001) 6:631–40. 10.1046/j.1365-2443.2001.00448.x 11473581

[23] Kasahara T Hooks JJ Dougherty SF Oppenheim JJ. Interleukin 2-mediated immune interferon (IFN-gamma) production by human T cells and T cell subsets. J Immunol (1983) 130:1784–9. 6403613

[24] Mosmann TR Cherwinski H Bond MW Giedlin MA Coffman RL. Two types of murine helper T cell clone. I. Definition according to profiles of lymphokine activities and secreted proteins. J Immunol (1986) 136:2348–57. 2419430

[25] Corthay A Skovseth DK Lundin KU Rosjo E Omholt H Hofgaard PO Primary antitumor immune response mediated by CD4+ T cells. Immunity (2005) 22:371–83. 10.1016/j.immuni.2005.02.003 15780993

[26] Matsushita H Hosoi A Ueha S Abe J Fujieda N Tomura M Cytotoxic T lymphocytes block tumor growth both by lytic activity and IFNgamma-dependent cell-cycle arrest. Cancer Immunol Res (2015) 3:26–36. 10.1158/2326-6066.CIR-14-0098 25127875

[27] Girardi M Oppenheim DE Steele CR Lewis JM Glusac E Filler R Regulation of cutaneous malignancy by gammadelta T cells. Science (2001) 294:605–9. 10.1126/science.1063916 11567106

[28] Gao Y Yang W Pan M Scully E Girardi M Augenlicht LH Gamma delta T cells provide an early source of interferon gamma in tumor immunity. J Exp Med (2003) 198:433–42. 10.1084/jem.20030584 12900519 PMC2194096

[29] Girardi M Glusac E Filler RB Roberts SJ Propperova I Lewis J The distinct contributions of murine T cell receptor (TCR)gammadelta+ and TCRalphabeta+ T cells to different stages of chemically induced skin cancer. J Exp Med (2003) 198:747–55. 10.1084/jem.20021282 12953094 PMC2194182

[30] Ribot JC deBarros A Pang DJ Neves JF Peperzak V Roberts SJ CD27 is a thymic determinant of the balance between interferon-gamma- and interleukin 17-producing gammadelta T cell subsets. Nat Immunol (2009) 10:427–36. 10.1038/ni.1717 19270712 PMC4167721

[31] Lanca T Costa MF Goncalves-Sousa N Rei M Grosso AR Penido C Protective role of the inflammatory CCR2/CCL2 chemokine pathway through recruitment of type 1 cytotoxic gammadelta T lymphocytes to tumor beds. J Immunol (2013) 190:6673–80. 10.4049/jimmunol.1300434 23686489

[32] Schmolka N Serre K Grosso AR Rei M Pennington DJ Gomes AQ Epigenetic and transcriptional signatures of stable versus plastic differentiation of proinflammatory gammadelta T cell subsets. Nat Immunol (2013) 14:1093–100. 10.1038/ni.2702 23995235 PMC4834994

[33] Silva-Santos B Serre K Norell H gammadelta T cells in cancer. Nat Rev Immunol (2015) 15:683–91. 10.1038/nri3904 26449179

[34] Yu J Wei M Becknell B Trotta R Liu S Boyd Z Pro- and antiinflammatory cytokine signaling: reciprocal antagonism regulates interferon-gamma production by human natural killer cells. Immunity (2006) 24:575–90. 10.1016/j.immuni.2006.03.016 16713975

[35] Keppel MP Saucier N Mah AY Vogel TP Cooper MA Activation-specific metabolic requirements for NK cell IFN-gamma production. J Immunol (2015) 194:1954–62. 10.4049/jimmunol.1402099 25595780 PMC4323953

[36] Yoshimoto T Takeda K Tanaka T Ohkusu K Kashiwamura S Okamura H IL-12 up-regulates IL-18 receptor expression on T cells, Th1 cells, and B cells: synergism with IL-18 for IFN-gamma production. J Immunol (1998) 161:3400–7. 9759857

[37] Flaishon L Hershkoviz R Lantner F Lider O Alon R Levo Y Autocrine secretion of interferon gamma negatively regulates homing of immature B cells. J Exp Med (2000) 192:1381–8. 10.1084/jem.192.9.1381 11067886 PMC2193359

[38] Barr TA Brown S Mastroeni P Gray D. TLR and B cell receptor signals to B cells differentially program primary and memory Th1 responses to *Salmonella enterica*. J Immunol (2010) 185:2783–9. 10.4049/jimmunol.1001431 20675594 PMC3745605

[39] Bao Y Liu X Han C Xu S Xie B Zhang Q Identification of IFN-gamma-producing innate B cells. Cell Res (2014) 24:161–76. 10.1038/cr.2013.155 24296781 PMC3915900

[40] Ohteki T Fukao T Suzue K Maki C Ito M Nakamura M Interleukin 12-dependent interferon gamma production by CD8alpha+ lymphoid dendritic cells. J Exp Med (1999) 189:1981–6. 10.1084/jem.189.12.1981 10377194 PMC2192968

[41] Zaidi MR Davis S Noonan FP Graff-Cherry C Hawley TS Walker RL Interferon-gamma links ultraviolet radiation to melanomagenesis in mice. Nature (2011) 469:548–53. 10.1038/nature09666 21248750 PMC3140101

[42] Darwich L Coma G Pena R Bellido R Blanco EJ Este JA Secretion of interferon-gamma by human macrophages demonstrated at the single-cell level after costimulation with interleukin (IL)-12 plus IL-18. Immunology (2009) 126:386–93. 10.1111/j.1365-2567.2008.02905.x 18759749 PMC2669819

[43] Chan SH Perussia B Gupta JW Kobayashi M Pospisil M Young HA Induction of interferon gamma production by natural killer cell stimulatory factor: characterization of the responder cells and synergy with other inducers. J Exp Med (1991) 173:869–79. 10.1084/jem.173.4.869 1672545 PMC2190821

[44] Ye J Ortaldo JR Conlon K Winkler-Pickett R Young HA. Cellular and molecular mechanisms of IFN-gamma production induced by IL-2 and IL-12 in a human NK cell line. J Leukoc Biol (1995) 58:225–33. 10.1002/jlb.58.2.225 7643015

[45] Carson WE Ross ME Baiocchi RA Marien MJ Boiani N Grabstein K Endogenous production of interleukin 15 by activated human monocytes is critical for optimal production of interferon-gamma by natural killer cells in vitro. J Clin Invest (1995) 96:2578–82. 10.1172/JCI118321 8675621 PMC185961

[46] Okamura H Tsutsi H Komatsu T Yutsudo M Hakura A Tanimoto T Cloning of a new cytokine that induces IFN-gamma production by T cells. Nature (1995) 378:88–91. 10.1038/378088a0 7477296

[47] Takeda K Tsutsui H Yoshimoto T Adachi O Yoshida N Kishimoto T Defective NK cell activity and Th1 response in IL-18-deficient mice. Immunity (1998) 8:383–90. 10.1016/S1074-7613(00)80543-9 9529155

[48] Nguyen KB Cousens LP Doughty LA Pien GC Durbin JE Biron CA. Interferon alpha/beta-mediated inhibition and promotion of interferon gamma: STAT1 resolves a paradox. Nat Immunol (2000) 1:70–6. 10.1038/76940 10881178

[49] Matikainen S Paananen A Miettinen M Kurimoto M Timonen T Julkunen I IFN-alpha and IL-18 synergistically enhance IFN-gamma production in human NK cells: differential regulation of Stat4 activation and IFN-gamma gene expression by IFN-alpha and IL-12. Eur J Immunol (2001) 31:2236–45. 10.1002/1521-4141(200107)31:7<2236::AID-IMMU2236>3.0.CO;2-G 11449378

[50] de Veer MJ Holko M Frevel M Walker E Der S Paranjape JM Functional classification of interferon-stimulated genes identified using microarrays. J Leukoc Biol (2001) 69:912–20. 10.1189/jlb.69.6.912 11404376

[51] Munder M Mallo M Eichmann K Modolell M. Murine macrophages secrete interferon gamma upon combined stimulation with interleukin (IL)-12 and IL-18: a novel pathway of autocrine macrophage activation. J Exp Med (1998) 187:2103–8. 10.1084/jem.187.12.2103 9625771 PMC2212367

[52] Rothfuchs AG Gigliotti D Palmblad K Andersson U Wigzell H Rottenberg ME. IFN-alpha beta-dependent, IFN-gamma secretion by bone marrow-derived macrophages controls an intracellular bacterial infection. J Immunol (2001) 167:6453–61. 10.4049/jimmunol.167.11.6453 11714812

[53] Fricke I Mitchell D Mittelstadt J Lehan N Heine H Goldmann T Mycobacteria induce IFN-gamma production in human dendritic cells via triggering of TLR2. J Immunol (2006) 176:5173–82. 10.4049/jimmunol.176.9.5173 16621981

[54] Tovey MG Streuli M Gresser I Gugenheim J Blanchard B Guymarho J Interferon messenger RNA is produced constitutively in the organs of normal individuals. Proc Natl Acad Sci U S A (1987) 84:5038–42. 10.1073/pnas.84.14.5038 3110782 PMC305242

[55] Chen HM Tanaka N Mitani Y Oda E Nozawa H Chen JZ Critical role for constitutive type I interferon signaling in the prevention of cellular transformation. Cancer Sci (2009) 100:449–56. 10.1111/j.1349-7006.2008.01051.x 19076978 PMC11158082

[56] Gattass CR King LB Luster AD Ashwell JD. Constitutive expression of interferon gamma-inducible protein 10 in lymphoid organs and inducible expression in T cells and thymocytes. J Exp Med (1994) 179:1373–8. 10.1084/jem.179.4.1373 8145049 PMC2191433

[57] Sercan O Hammerling GJ Arnold B Schuler T. Innate immune cells contribute to the IFN-gamma-dependent regulation of antigen-specific CD8+ T cell homeostasis. J Immunol (2006) 176:735–9. 10.4049/jimmunol.176.2.735 16393956

[58] Baldridge MT King KY Boles NC Weksberg DC Goodell MA. Quiescent haematopoietic stem cells are activated by IFN-gamma in response to chronic infection. Nature (2010) 465:793–7. 10.1038/nature09135 20535209 PMC2935898

[59] Duque G Huang DC Dion N Macoritto M Rivas D Li W Interferon-gamma plays a role in bone formation in vivo and rescues osteoporosis in ovariectomized mice. J Bone Miner Res (2011) 26:1472–83. 10.1002/jbmr.350 21308779

[60] Karupiah G Xie QW Buller RM Nathan C Duarte C MacMicking JD. Inhibition of viral replication by interferon-gamma-induced nitric oxide synthase. Science (1993) 261:1445–8. 10.1126/science.7690156 7690156

[61] Shrestha B Wang T Samuel MA Whitby K Craft J Fikrig E Gamma interferon plays a crucial early antiviral role in protection against West Nile virus infection. J Virol (2006) 80:5338–48. 10.1128/JVI.00274-06 16699014 PMC1472130

[62] Ferber IA Brocke S Taylor-Edwards C Ridgway W Dinisco C Steinman L Mice with a disrupted IFN-gamma gene are susceptible to the induction of experimental autoimmune encephalomyelitis (EAE). J Immunol (1996) 156:5–7. 8598493

[63] Sosa RA Murphey C Robinson RR Forsthuber TG IFN-gamma ameliorates autoimmune encephalomyelitis by limiting myelin lipid peroxidation. Proc Natl Acad Sci U S A (2015) 112:E5038–47. 10.1073/pnas.1505955112 26305941 PMC4568689

[64] Lee SJ Jang BC Lee SW Yang YI Suh SI Park YM Interferon regulatory factor-1 is prerequisite to the constitutive expression and IFN-gamma-induced upregulation of B7-H1 (CD274). FEBS Lett (2006) 580:755–62. 10.1016/j.febslet.2005.12.093 16413538

[65] Nakajima C Uekusa Y Iwasaki M Yamaguchi N Mukai T Gao P A role of interferon-gamma (IFN-gamma) in tumor immunity: T cells with the capacity to reject tumor cells are generated but fail to migrate to tumor sites in IFN-gamma-deficient mice. Cancer Res (2001) 61:3399–405. 11309299

[66] Bernabei P Coccia EM Rigamonti L Bosticardo M Forni G Pestka S Interferon-gamma receptor 2 expression as the deciding factor in human T, B, and myeloid cell proliferation or death. J Leukoc Biol (2001) 70:950–60. 10.1189/jlb.70.6.950 11739558

[67] Pernis A Gupta S Gollob KJ Garfein E Coffman RL Schindler C Lack of interferon gamma receptor beta chain and the prevention of interferon gamma signaling in TH1 cells. Science (1995) 269:245–7. 10.1126/science.7618088 7618088

[68] Bach EA Szabo SJ Dighe AS Ashkenazi A Aguet M Murphy KM Ligand-induced autoregulation of IFN-gamma receptor beta chain expression in T helper cell subsets. Science (1995) 270:1215–8. 10.1126/science.270.5239.1215 7502050

[69] Szabo SJ Kim ST Costa GL Zhang X Fathman CG Glimcher LH. A novel transcription factor, T-bet, directs Th1 lineage commitment. Cell (2000) 100:655–69. 10.1016/S0092-8674(00)80702-3 10761931

[70] Bradley LM Dalton DK Croft M A direct role for IFN-gamma in regulation of Th1 cell development. J Immunol (1996) 157:1350–8. 8759714

[71] Girdlestone J Wing M. Autocrine activation by interferon-gamma of STAT factors following T cell activation. Eur J Immunol (1996) 26:704–9. 10.1002/eji.1830260329 8605941

[72] Lighvani AA Frucht DM Jankovic D Yamane H Aliberti J Hissong BD T-bet is rapidly induced by interferon-gamma in lymphoid and myeloid cells. Proc Natl Acad Sci U S A (2001) 98:15137–42. 10.1073/pnas.261570598 11752460 PMC64996

[73] Maggi E Parronchi P Manetti R Simonelli C Piccinni MP Rugiu FS Reciprocal regulatory effects of IFN-gamma and IL-4 on the in vitro development of human Th1 and Th2 clones. J Immunol (1992) 148:2142–7. 1532000

[74] Halonen SK Woods T McInnerney K Weiss LM. Microarray analysis of IFN-gamma response genes in astrocytes. J Neuroimmunol (2006) 175:19–30. 10.1016/j.jneuroim.2006.02.015 16631260 PMC3109620

[75] Rock RB Hu S Deshpande A Munir S May BJ Baker CA Transcriptional response of human microglial cells to interferon-gamma. Genes Immun (2005) 6:712–9. 10.1038/sj.gene.6364246 16163375

[76] Majoros A Platanitis E Kernbauer-Holzl E Rosebrock F Muller M Decker T. Canonical and non-canonical aspects of JAK-STAT signaling: lessons from interferons for cytokine responses. Front Immunol (2017) 8:29. 10.3389/fimmu.2017.00029 28184222 PMC5266721

[77] Greenlund AC Farrar MA Viviano BL Schreiber RD. Ligand-induced IFN gamma receptor tyrosine phosphorylation couples the receptor to its signal transduction system (p91). EMBO J (1994) 13:1591–600. 8156998 10.1002/j.1460-2075.1994.tb06422.x PMC394989

[78] Decker T Lew DJ Mirkovitch J Darnell JE Jr. Cytoplasmic activation of GAF, an IFN-gamma-regulated DNA-binding factor. EMBO J (1991) 10:927–32. 1901265 10.1002/j.1460-2075.1991.tb08026.x PMC452736

[79] Chatterjee-Kishore M Wright KL Ting JP Stark GR. How Stat1 mediates constitutive gene expression: a complex of unphosphorylated Stat1 and IRF1 supports transcription of the LMP2 gene. EMBO J (2000) 19:4111–22. 10.1093/emboj/19.15.4111 10921891 PMC306607

[80] Rettino A Clarke NM. Genome-wide identification of IRF1 binding sites reveals extensive occupancy at cell death associated genes. J Carcinog Mutagen (2013) S6-009:2157–518. 10.4172/2157-2518.S6-009 25893139 PMC4398980

[81] Murtas D Maric D De Giorgi V Reinboth J Worschech A Fetsch P IRF-1 responsiveness to IFN-gamma predicts different cancer immune phenotypes. Br J Cancer (2013) 109:76–82. 10.1038/bjc.2013.335 23807161 PMC3708578

[82] Ulloa L Doody J Massague J. Inhibition of transforming growth factor-beta/SMAD signalling by the interferon-gamma/STAT pathway. Nature (1999) 397:710–3. 10.1038/17826 10067896

[83] Ramana CV Grammatikakis N Chernov M Nguyen H Goh KC Williams BR Regulation of c-myc expression by IFN-gamma through Stat1-dependent and -independent pathways. EMBO J (2000) 19:263–72. 10.1093/emboj/19.2.263 10637230 PMC305560

[84] Chin YE Kitagawa M Su WC You ZH Iwamoto Y Fu XY. Cell growth arrest and induction of cyclin-dependent kinase inhibitor p21 WAF1/CIP1 mediated by STAT1. Science (1996) 272:719–22. 10.1126/science.272.5262.719 8614832

[85] Xing S Wanting TH Zhao W Ma J Wang S Xu X Transgenic expression of JAK2V617F causes myeloproliferative disorders in mice. Blood (2008) 111:5109–17. 10.1182/blood-2007-05-091579 18334677 PMC2384138

[86] Flex E Petrangeli V Stella L Chiaretti S Hornakova T Knoops L Somatically acquired JAK1 mutations in adult acute lymphoblastic leukemia. J Exp Med (2008) 205:751–8. 10.1084/jem.20072182 18362173 PMC2292215

[87] Bottos A Gotthardt D Gill JW Gattelli A Frei A Tzankov A Decreased NK-cell tumour immunosurveillance consequent to JAK inhibition enhances metastasis in breast cancer models. Nat Commun (2016) 7:12258. 10.1038/ncomms12258 27406745 PMC4947169

[88] Yu H Pardoll D Jove R. STATs in cancer inflammation and immunity: a leading role for STAT3. Nat Rev Cancer (2009) 9:798–809. 10.1038/nrc2734 19851315 PMC4856025

[89] Messina NL Banks KM Vidacs E Martin BP Long F Christiansen AJ Modulation of antitumour immune responses by intratumoural Stat1 expression. Immunol Cell Biol (2013) 91:556–67. 10.1038/icb.2013.41 23958683

[90] Fallarino F Gajewski TF. Cutting edge: differentiation of antitumor CTL in vivo requires host expression of Stat1. J Immunol (1999) 163:4109–13. 10510345

[91] Chan SR Vermi W Luo J Lucini L Rickert C Fowler AM STAT1-deficient mice spontaneously develop estrogen receptor alpha-positive luminal mammary carcinomas. Breast Cancer Res (2012) 14:R16 10.1186/bcr3100 22264274 PMC3496133

[92] Andrianifahanana M Singh AP Nemos C Ponnusamy MP Moniaux N Mehta PP IFN-gamma-induced expression of MUC4 in pancreatic cancer cells is mediated by STAT-1 upregulation: a novel mechanism for IFN-gamma response. Oncogene (2007) 26:7251–61. 10.1038/sj.onc.1210532 17525742

[93] Hix LM Karavitis J Khan MW Shi YH Khazaie K Zhang M. Tumor STAT1 transcription factor activity enhances breast tumor growth and immune suppression mediated by myeloid-derived suppressor cells. J Biol Chem (2013) 288:11676–88. 10.1074/jbc.M112.441402 23486482 PMC3636858

[94] Weichselbaum RR Ishwaran H Yoon T Nuyten DS Baker SW Khodarev N An interferon-related gene signature for DNA damage resistance is a predictive marker for chemotherapy and radiation for breast cancer. Proc Natl Acad Sci U S A (2008) 105:18490–5. 10.1073/pnas.0809242105 19001271 PMC2587578

[95] Khodarev NN Roach P Pitroda SP Golden DW Bhayani M Shao MY STAT1 pathway mediates amplification of metastatic potential and resistance to therapy. PLoS One (2009) 4:e5821. 10.1371/journal.pone.0005821 19503789 PMC2688034

[96] Jauch D Martin M Schiechl G Kesselring R Schlitt HJ Geissler EK Interleukin 21 controls tumour growth and tumour immunosurveillance in colitis-associated tumorigenesis in mice. Gut (2011) 60:1678–86. 10.1136/gutjnl-2011-300612 21948944

[97] Carbotti G Barisione G Airoldi I Mezzanzanica D Bagnoli M Ferrero S IL-27 induces the expression of IDO and PD-L1 in human cancer cells. Oncotarget (2015) 6:43267–80. 10.18632/oncotarget.6530 26657115 PMC4791231

[98] Turnis ME Sawant DV Szymczak-Workman AL Andrews LP Delgoffe GM Yano H Interleukin-35 limits anti-tumor immunity. Immunity (2016) 44:316–29. 10.1016/j.immuni.2016.01.013 26872697 PMC4758699

[99] Huang C Li N Li Z Chang A Chen Y Zhao T Tumour-derived Interleukin 35 promotes pancreatic ductal adenocarcinoma cell extravasation and metastasis by inducing ICAM1 expression. Nat Commun (2017) 8:14035. 10.1038/ncomms14035 28102193 PMC5253665

[100] Zhao Z Chen X Hao S Jia R Wang N Chen S Increased interleukin-35 expression in tumor-infiltrating lymphocytes correlates with poor prognosis in patients with breast cancer. Cytokine (2017) 89:76–81. 10.1016/j.cyto.2016.09.012 27681506

[101] Zou JM Qin J Li YC Wang Y Li D Shu Y IL-35 induces N2 phenotype of neutrophils to promote tumor growth. Oncotarget (2017) 8:33501–14. 10.18632/oncotarget.16819 28432279 PMC5464885

[102] Endo TA Masuhara M Yokouchi M Suzuki R Sakamoto H Mitsui K A new protein containing an SH2 domain that inhibits JAK kinases. Nature (1997) 387:921–4. 10.1038/43213 9202126

[103] Starr R Willson TA Viney EM Murray LJ Rayner JR Jenkins BJ A family of cytokine-inducible inhibitors of signalling. Nature (1997) 387:917–21. 10.1038/43206 9202125

[104] Zhang JG Farley A Nicholson SE Willson TA Zugaro LM Simpson RJ The conserved SOCS box motif in suppressors of cytokine signaling binds to elongins B and C and may couple bound proteins to proteasomal degradation. Proc Natl Acad Sci U S A (1999) 96:2071–6. 10.1073/pnas.96.5.2071 10051596 PMC26738

[105] Kamizono S Hanada T Yasukawa H Minoguchi S Kato R Minoguchi M The SOCS box of SOCS-1 accelerates ubiquitin-dependent proteolysis of TEL-JAK2. J Biol Chem (2001) 276:12530–8. 10.1074/jbc.M010074200 11278610

[106] Chang JH Xiao Y Hu H Jin J Yu J Zhou X Ubc13 maintains the suppressive function of regulatory T cells and prevents their conversion into effector-like T cells. Nat Immunol (2012) 13:481–90. 10.1038/ni.2267 22484734 PMC3361639

[107] Kim WS Kim MJ Kim DO Byun JE Huy H Song HY Suppressor of cytokine signaling 2 negatively regulates NK cell differentiation by inhibiting JAK2 activity. Sci Rep (2017) 7:46153. 10.1038/srep46153 28383049 PMC5382670

[108] Choudhury GG. A linear signal transduction pathway involving phosphatidylinositol 3-kinase, protein kinase Cepsilon, and MAPK in mesangial cells regulates interferon-gamma-induced STAT1alpha transcriptional activation. J Biol Chem (2004) 279:27399–409. 10.1074/jbc.M403530200 15082710

[109] Qing Y Stark GR Alternative activation of STAT1 and STAT3 in response to interferon-gamma. J Biol Chem (2004) 279:41679–85. 10.1074/jbc.M406413200 15284232

[110] Meinke A Barahmand-Pour F Wohrl S Stoiber D Decker T. Activation of different Stat5 isoforms contributes to cell-type-restricted signaling in response to interferons. Mol Cell Biol (1996) 16:6937–44. 10.1128/MCB.16.12.6937 8943349 PMC231697

[111] Deb A Haque SJ Mogensen T Silverman RH Williams BR. RNA-dependent protein kinase PKR is required for activation of NF-kappa B by IFN-gamma in a STAT1-independent pathway. J Immunol (2001) 166:6170–80. 10.4049/jimmunol.166.10.6170 11342638

[112] Lewis M Amento EP Unemori EN. Transcriptional inhibition of stromelysin by interferon-gamma in normal human fibroblasts is mediated by the AP-1 domain. J Cell Biochem (1999) 72:373–86. 10.1002/(SICI)1097-4644(19990301)72:3<373::AID-JCB7>3.0.CO;2-N 10022519

[113] Shirayoshi Y Burke PA Appella E Ozato K. Interferon-induced transcription of a major histocompatibility class I gene accompanies binding of inducible nuclear factors to the interferon consensus sequence. Proc Natl Acad Sci U S A (1988) 85:5884–8. 10.1073/pnas.85.16.5884 2457903 PMC281869

[114] Amaldi I Reith W Berte C Mach B. Induction of HLA class II genes by IFN-gamma is transcriptional and requires a trans-acting protein. J Immunol (1989) 142:999–1004. 2492334

[115] Belich MP Glynne RJ Senger G Sheer D Trowsdale J. Proteasome components with reciprocal expression to that of the MHC-encoded LMP proteins. Curr Biol (1994) 4:769–76. 10.1016/S0960-9822(00)00174-3 7820546

[116] Nandi D Jiang H Monaco JJ. Identification of MECL-1 (LMP-10) as the third IFN-gamma-inducible proteasome subunit. J Immunol (1996) 156:2361–4. 8786291

[117] Cramer LA Nelson SL Klemsz MJ. Synergistic induction of the Tap-1 gene by IFN-gamma and lipopolysaccharide in macrophages is regulated by STAT1. J Immunol (2000) 165:3190–7. 10.4049/jimmunol.165.6.3190 10975834

[118] Johnson DR Pober JS. Tumor necrosis factor and immune interferon synergistically increase transcription of HLA class I heavy- and light-chain genes in vascular endothelium. Proc Natl Acad Sci U S A (1990) 87:5183–7. 10.1073/pnas.87.13.5183 2164225 PMC54286

[119] Seliger B Schreiber K Delp K Meissner M Hammers S Reichert T Downregulation of the constitutive tapasin expression in human tumor cells of distinct origin and its transcriptional upregulation by cytokines. Tissue Antigens (2001) 57:39–45. 10.1034/j.1399-0039.2001.057001039.x 11169257

[120] Kern I Steimle V Siegrist CA Mach B. The two novel MHC class II transactivators RFX5 and CIITA both control expression of HLA-DM genes. Int Immunol (1995) 7:1295–9. 10.1093/intimm/7.8.1295 7495736

[121] Lah TT Hawley M Rock KL Goldberg AL. Gamma-interferon causes a selective induction of the lysosomal proteases, cathepsins B and L, in macrophages. FEBS Lett (1995) 363:85–9. 10.1016/0014-5793(95)00287-J 7729559

[122] Steimle V Siegrist CA Mottet A Lisowska-Grospierre B Mach B. Regulation of MHC class II expression by interferon-gamma mediated by the transactivator gene CIITA. Science (1994) 265:106–9. 10.1126/science.8016643 8016643

[123] Zhao M Flynt FL Hong M Chen H Gilbert CA Briley NT MHC class II transactivator (CIITA) expression is upregulated in multiple myeloma cells by IFN-gamma. Mol Immunol (2007) 44:2923–32. 10.1016/j.molimm.2007.01.009 17300840 PMC1892219

[124] Deffrennes V Vedrenne J Stolzenberg MC Piskurich J Barbieri G Ting JP Constitutive expression of MHC class II genes in melanoma cell lines results from the transcription of class II transactivator abnormally initiated from its B cell-specific promoter. J Immunol (2001) 167:98–106. 10.4049/jimmunol.167.1.98 11418637

[125] Maraskovsky E Chen WF Shortman K. IL-2 and IFN-gamma are two necessary lymphokines in the development of cytolytic T cells. J Immunol (1989) 143:1210–4. 2501391

[126] Curtsinger JM Agarwal P Lins DC Mescher MF Autocrine IFN-gamma promotes naive CD8 T cell differentiation and synergizes with IFN-alpha to stimulate strong function. J Immunol (2012) 189:659–68. 10.4049/jimmunol.1102727 22706089 PMC3392455

[127] Akbar SM Inaba K Onji M. Upregulation of MHC class II antigen on dendritic cells from hepatitis B virus transgenic mice by interferon-gamma: abrogation of immune response defect to a T-cell-dependent antigen. Immunology (1996) 87:519–27. 10.1046/j.1365-2567.1996.516576.x 8675204 PMC1384128

[128] Walter W Lingnau K Schmitt E Loos M Maeurer MJ. MHC class II antigen presentation pathway in murine tumours: tumour evasion from immunosurveillance? Br J Cancer (2000) 83:1192–201. 10.1054/bjoc.2000.1415 11027433 PMC2363595

[129] Haabeth OA Lorvik KB Hammarstrom C Donaldson IM Haraldsen G Bogen B Inflammation driven by tumour-specific Th1 cells protects against B-cell cancer. Nat Commun (2011) 2:240. 10.1038/ncomms1239 21407206 PMC3072106

[130] Nathan CF Murray HW Wiebe ME Rubin BY. Identification of interferon-gamma as the lymphokine that activates human macrophage oxidative metabolism and antimicrobial activity. J Exp Med (1983) 158:670–89. 10.1084/jem.158.3.670 6411853 PMC2187114

[131] Sadlik JR Hoyer M Leyko MA Horvat R Parmely M Whitacre C Lymphocyte supernatant-induced human monocyte tumoricidal activity: dependence on the presence of gamma-interferon. Cancer Res (1985) 45:1940–5. 3921233

[132] Taniyama T Taki S Akiyama Y Yoshizawa K Hamuro J Arai K Constitutive production of novel macrophage-activating factor(s) by human T cell hybridomas. Clin Invest Med (1990) 13:305–12. 2127737

[133] Higuchi M Sugimoto M Kobayashi Y Osawa T. Human macrophage-activating factors for cytotoxicity. I. Establishment of a human T-cell hybridoma that produces macrophage-activating factors for cytotoxicity. Microbiol Immunol (1987) 31:469–79. 10.1111/j.1348-0421.1987.tb03109.x 3116371

[134] Waddell SJ Popper SJ Rubins KH Griffiths MJ Brown PO Levin M Dissecting interferon-induced transcriptional programs in human peripheral blood cells. PLoS One (2010) 5:e9753. 10.1371/journal.pone.0009753 20339534 PMC2842296

[135] Shiloh MU MacMicking JD Nicholson S Brause JE Potter S Marino M Phenotype of mice and macrophages deficient in both phagocyte oxidase and inducible nitric oxide synthase. Immunity (1999) 10:29–38. 10.1016/S1074-7613(00)80004-7 10023768

[136] Pfefferkorn ER. Interferon gamma blocks the growth of *Toxoplasma gondii* in human fibroblasts by inducing the host cells to degrade tryptophan. Proc Natl Acad Sci U S A (1984) 81:908–12. 10.1073/pnas.81.3.908 6422465 PMC344948

[137] Pak-Wittel MA Yang L Sojka DK Rivenbark JG Yokoyama WM Interferon-gamma mediates chemokine-dependent recruitment of natural killer cells during viral infection. Proc Natl Acad Sci U S A (2013) 110:E50–9. 10.1073/pnas.1220456110 23248310 PMC3538256

[138] Valledor AF Sanchez-Tillo E Arpa L Park JM Caelles C Lloberas J Selective roles of MAPKs during the macrophage response to IFN-gamma. J Immunol (2008) 180:4523–9. 10.4049/jimmunol.180.7.4523 18354174

[139] Zhang Y Apilado R Coleman J Ben-Sasson S Tsang S Hu-Li J Interferon gamma stabilizes the T helper cell type 1 phenotype. J Exp Med (2001) 194:165–72. 10.1084/jem.194.2.165 11457891 PMC2193457

[140] Maldonado RA Irvine DJ Schreiber R Glimcher LH. A role for the immunological synapse in lineage commitment of CD4 lymphocytes. Nature (2004) 431:527–32. 10.1038/nature02916 15386021

[141] Schulz EG Mariani L Radbruch A Hofer T. Sequential polarization and imprinting of type 1 T helper lymphocytes by interferon-gamma and interleukin-12. Immunity (2009) 30:673–83. 10.1016/j.immuni.2009.03.013 19409816

[142] Oriss TB McCarthy SA Morel BF Campana MA Morel PA. Crossregulation between T helper cell (Th)1 and Th2: inhibition of Th2 proliferation by IFN-gamma involves interference with IL-1. J Immunol (1997) 158:3666–72. 9103429

[143] Gajewski TF Fitch FW. Anti-proliferative effect of IFN-gamma in immune regulation. I. IFN-gamma inhibits the proliferation of Th2 but not Th1 murine helper T lymphocyte clones. J Immunol (1988) 140:4245–52. 2967332

[144] Naka T Tsutsui H Fujimoto M Kawazoe Y Kohzaki H Morita Y SOCS-1/SSI-1-deficient NKT cells participate in severe hepatitis through dysregulated cross-talk inhibition of IFN-gamma and IL-4 signaling in vivo. Immunity (2001) 14:535–45. 10.1016/S1074-7613(01)00132-7 11371356

[145] Yu CR Mahdi RM Ebong S Vistica BP Chen J Guo Y Cell proliferation and STAT6 pathways are negatively regulated in T cells by STAT1 and suppressors of cytokine signaling. J Immunol (2004) 173:737–46. 10.4049/jimmunol.173.2.737 15240659

[146] Hwang ES Szabo SJ Schwartzberg PL Glimcher LH. T helper cell fate specified by kinase-mediated interaction of T-bet with GATA-3. Science (2005) 307:430–3. 10.1126/science.1103336 15662016

[147] Höfer T Nathansen H Lohning M Radbruch A Heinrich R. GATA-3 transcriptional imprinting in Th2 lymphocytes: a mathematical model. Proc Natl Acad Sci U S A (2002) 99:9364–8. 10.1073/pnas.142284699 12087127 PMC123146

[148] Kurata H Lee HJ O’Garra A Arai N. Ectopic expression of activated Stat6 induces the expression of Th2-specific cytokines and transcription factors in developing Th1 cells. Immunity (1999) 11:677–88. 10.1016/S1074-7613(00)80142-9 10626890

[149] Ouyang W Lohning M Gao Z Assenmacher M Ranganath S Radbruch A Stat6-independent GATA-3 autoactivation directs IL-4-independent Th2 development and commitment. Immunity (2000) 12:27–37. 10.1016/S1074-7613(00)80156-9 10661403

[150] Dickensheets HL Donnelly RP. Inhibition of IL-4-inducible gene expression in human monocytes by type I and type II interferons. J Leukoc Biol (1999) 65:307–12. 10.1002/jlb.65.3.307 10080532

[151] Kacha AK Fallarino F Markiewicz MA Gajewski TF. Cutting edge: spontaneous rejection of poorly immunogenic P1.HTR tumors by Stat6-deficient mice. J Immunol (2000) 165:6024–8. 10.4049/jimmunol.165.11.6024 11086033

[152] Harrington LE Hatton RD Mangan PR Turner H Murphy TL Murphy KM Interleukin 17-producing CD4+ effector T cells develop via a lineage distinct from the T helper type 1 and 2 lineages. Nat Immunol (2005) 6:1123–32. 10.1038/ni1254 16200070

[153] Park H Li Z Yang XO Chang SH Nurieva R Wang YH A distinct lineage of CD4 T cells regulates tissue inflammation by producing interleukin 17. Nat Immunol (2005) 6:1133–41. 10.1038/ni1261 16200068 PMC1618871

[154] Kelchtermans H Schurgers E Geboes L Mitera T Van Damme J Van Snick J Effector mechanisms of interleukin-17 in collagen-induced arthritis in the absence of interferon-gamma and counteraction by interferon-gamma. Arthritis Res Ther (2009) 11:R122. 10.1186/ar2787 19686583 PMC2745806

[155] Kimura A Naka T Nohara K Fujii-Kuriyama Y Kishimoto T. Aryl hydrocarbon receptor regulates Stat1 activation and participates in the development of Th17 cells. Proc Natl Acad Sci U S A (2008) 105:9721–6. 10.1073/pnas.0804231105 18607004 PMC2474493

[156] Tanaka K Ichiyama K Hashimoto M Yoshida H Takimoto T Takaesu G Loss of suppressor of cytokine signaling 1 in helper T cells leads to defective Th17 differentiation by enhancing antagonistic effects of IFN-gamma on STAT3 and Smads. J Immunol (2008) 180:3746–56. 10.4049/jimmunol.180.6.3746 18322180

[157] Herrero C Hu X Li WP Samuels S Sharif MN Kotenko S Reprogramming of IL-10 activity and signaling by IFN-gamma. J Immunol (2003) 171:5034–41. 10.4049/jimmunol.171.10.5034 14607900

[158] Lazarevic V Chen X Shim JH Hwang ES Jang E Bolm AN T-bet represses T(H)17 differentiation by preventing Runx1-mediated activation of the gene encoding RORgammat. Nat Immunol (2011) 12:96–104. 10.1038/ni.1969 21151104 PMC3077962

[159] Barrios-Rodiles M Chadee K. Novel regulation of cyclooxygenase-2 expression and prostaglandin E2 production by IFN-gamma in human macrophages. J Immunol (1998) 161:2441–8. 9725242

[160] Zhou M Zhang Y Ardans JA Wahl LM. Interferon-gamma differentially regulates monocyte matrix metalloproteinase-1 and -9 through tumor necrosis factor-alpha and caspase 8. J Biol Chem (2003) 278:45406–13. 10.1074/jbc.M309075200 12960156

[161] Irmler IM Gajda M Brauer R. Exacerbation of antigen-induced arthritis in IFN-gamma-deficient mice as a result of unrestricted IL-17 response. J Immunol (2007) 179:6228–36. 10.4049/jimmunol.179.9.6228 17947698

[162] Kelchtermans H Struyf S De Klerck B Mitera T Alen M Geboes L Protective role of IFN-gamma in collagen-induced arthritis conferred by inhibition of mycobacteria-induced granulocyte chemotactic protein-2 production. J Leukoc Biol (2007) 81:1044–53. 10.1189/jlb.0806486 17200147

[163] Hu X Paik PK Chen J Yarilina A Kockeritz L Lu TT IFN-gamma suppresses IL-10 production and synergizes with TLR2 by regulating GSK3 and CREB/AP-1 proteins. Immunity (2006) 24:563–74. 10.1016/j.immuni.2006.02.014 16713974

[164] Park IK Letterio JJ Gorham JD. TGF-beta 1 inhibition of IFN-gamma-induced signaling and Th1 gene expression in CD4+ T cells is Smad3 independent but MAP kinase dependent. Mol Immunol (2007) 44:3283–90. 10.1016/j.molimm.2007.02.024 17403540 PMC2134969

[165] Caretto D Katzman SD Villarino AV Gallo E Abbas AK. Cutting edge: the Th1 response inhibits the generation of peripheral regulatory T cells. J Immunol (2010) 184:30–4. 10.4049/jimmunol.0903412 19949064 PMC2908389

[166] Olalekan SA Cao Y Hamel KM Finnegan A B cells expressing IFN-gamma suppress Treg-cell differentiation and promote autoimmune experimental arthritis. Eur J Immunol (2015) 45:988–98. 10.1002/eji.201445036 25645456 PMC4438566

[167] Willenborg DO Fordham S Bernard CC Cowden WB Ramshaw IA. IFN-gamma plays a critical down-regulatory role in the induction and effector phase of myelin oligodendrocyte glycoprotein-induced autoimmune encephalomyelitis. J Immunol (1996) 157:3223–7. 8871615

[168] Nishibori T Tanabe Y Su L David M. Impaired development of CD4+ CD25+ regulatory T cells in the absence of STAT1: increased susceptibility to autoimmune disease. J Exp Med (2004) 199:25–34. 10.1084/jem.20020509 14699080 PMC1193645

[169] Wang Z Hong J Sun W Xu G Li N Chen X Role of IFN-gamma in induction of Foxp3 and conversion of CD4+ CD25- T cells to CD4+ Tregs. J Clin Invest (2006) 116:2434–41. 10.1172/JCI25826 16906223 PMC1533873

[170] Vermeire K Heremans H Vandeputte M Huang S Billiau A Matthys P. Accelerated collagen-induced arthritis in IFN-gamma receptor-deficient mice. J Immunol (1997) 158:5507–13. 9164974

[171] Wei B Baker S Wieckiewicz J Wood KJ. IFN-gamma triggered STAT1-PKB/AKT signalling pathway influences the function of alloantigen reactive regulatory T cells. Am J Transplant (2010) 10:69–80. 10.1111/j.1600-6143.2009.02858.x 19889125 PMC3158990

[172] Feng T Cao AT Weaver CT Elson CO Cong Y Interleukin-12 converts Foxp3+ regulatory T cells to interferon-gamma-producing Foxp3+ T cells that inhibit colitis. Gastroenterology (2011) 140:2031–43. 10.1053/j.gastro.2011.03.009 21419767 PMC3109200

[173] Sawitzki B Kingsley CI Oliveira V Karim M Herber M Wood KJ. IFN-gamma production by alloantigen-reactive regulatory T cells is important for their regulatory function in vivo. J Exp Med (2005) 201:1925–35. 10.1084/jem.20050419 15967822 PMC2212028

[174] Koenecke C Lee CW Thamm K Fohse L Schafferus M Mittrucker HW IFN-gamma production by allogeneic Foxp3+ regulatory T cells is essential for preventing experimental graft-versus-host disease. J Immunol (2012) 189:2890–6. 10.4049/jimmunol.1200413 22869903

[175] Greifenberg V Ribechini E Rossner S Lutz MB. Myeloid-derived suppressor cell activation by combined LPS and IFN-gamma treatment impairs DC development. Eur J Immunol (2009) 39:2865–76. 10.1002/eji.200939486 19637228

[176] Shime H Maruyama A Yoshida S Takeda Y Matsumoto M Seya T Toll-like receptor 2 ligand and interferon-gamma suppress anti-tumor T cell responses by enhancing the immunosuppressive activity of monocytic myeloid-derived suppressor cells. Oncoimmunology (2017) 7:e1373231 10.1080/2162402X.2017.1373231 29296526 PMC5739553

[177] Taylor MW Feng GS. Relationship between interferon-gamma, indoleamine 2,3-dioxygenase, and tryptophan catabolism. FASEB J (1991) 5:2516–22. 10.1096/fasebj.5.11.1907934 1907934

[178] Malone DG Dolan PW Brown RR Kalayoglu MV Arend RA Byrne GI Interferon gamma induced production of indoleamine 2,3 dioxygenase in cultured human synovial cells. J Rheumatol (1994) 21:1011–9. 7523670

[179] Jurgens B Hainz U Fuchs D Felzmann T Heitger A. Interferon-gamma-triggered indoleamine 2,3-dioxygenase competence in human monocyte-derived dendritic cells induces regulatory activity in allogeneic T cells. Blood (2009) 114:3235–43. 10.1182/blood-2008-12-195073 19625705

[180] Sarkar SA Wong R Hackl SI Moua O Gill RG Wiseman A Induction of indoleamine 2,3-dioxygenase by interferon-gamma in human islets. Diabetes (2007) 56:72–9. 10.2337/db06-0617 17192467

[181] Abiko K Matsumura N Hamanishi J Horikawa N Murakami R Yamaguchi K IFN-gamma from lymphocytes induces PD-L1 expression and promotes progression of ovarian cancer. Br J Cancer (2015) 112:1501–9. 10.1038/bjc.2015.101 25867264 PMC4453666

[182] Mimura K Teh JL Okayama H Shiraishi K Kua LF Koh V PD-L1 expression is mainly regulated by interferon gamma associated with JAK-STAT pathway in gastric cancer. Cancer Sci (2018) 109:43–53. 10.1111/cas.13424 29034543 PMC5765310

[183] Garcia-Diaz A Shin DS Moreno BH Saco J Escuin-Ordinas H Rodriguez GA Interferon receptor signaling pathways regulating PD-L1 and PD-L2 expression. Cell Rep (2017) 19:1189–201. 10.1016/j.celrep.2017.04.031 28494868 PMC6420824

[184] Beatty G Paterson Y. IFN-gamma-dependent inhibition of tumor angiogenesis by tumor-infiltrating CD4+ T cells requires tumor responsiveness to IFN-gamma. J Immunol (2001) 166:2276–82. 10.4049/jimmunol.166.4.2276 11160282

[185] Zhang R Banik NL Ray SK. Combination of all-trans retinoic acid and interferon-gamma suppressed PI3K/Akt survival pathway in glioblastoma T98G cells whereas NF-kappaB survival signaling in glioblastoma U87MG cells for induction of apoptosis. Neurochem Res (2007) 32:2194–202. 10.1007/s11064-007-9417-7 17616812

[186] Detjen KM Farwig K Welzel M Wiedenmann B Rosewicz S. Interferon gamma inhibits growth of human pancreatic carcinoma cells via caspase-1 dependent induction of apoptosis. Gut (2001) 49:251–62. 10.1136/gut.49.2.251 11454803 PMC1728385

[187] Kotredes KP Gamero AM. Interferons as inducers of apoptosis in malignant cells. J Interferon Cytokine Res (2013) 33:162–70. 10.1089/jir.2012.0110 23570382 PMC3624694

[188] Sucker A Zhao F Pieper N Heeke C Maltaner R Stadtler N Acquired IFNgamma resistance impairs anti-tumor immunity and gives rise to T-cell-resistant melanoma lesions. Nat Commun (2017) 8:15440 10.1038/ncomms15440 28561041 PMC5460020

[189] Heyman M Grander D Brondum-Nielsen K Cederblad B Liu Y Xu B Interferon system defects in malignant T-cells. Leukemia (1994) 8:425–34. 8127147

[190] Satoh A Toyota M Ikeda H Morimoto Y Akino K Mita H Epigenetic inactivation of class II transactivator (CIITA) is associated with the absence of interferon-gamma-induced HLA-DR expression in colorectal and gastric cancer cells. Oncogene (2004) 23:8876–86. 10.1038/sj.onc.1208144 15467734

[191] van den Elsen PJ Holling TM van der Stoep N Boss JM. DNA methylation and expression of major histocompatibility complex class I and class II transactivator genes in human developmental tumor cells and in T cell malignancies. Clin Immunol (2003) 109:46–52. 10.1016/S1521-6616(03)00200-6 14585275

[192] Yazawa T Kamma H Fujiwara M Matsui M Horiguchi H Satoh H Lack of class II transactivator causes severe deficiency of HLA-DR expression in small cell lung cancer. J Pathol (1999) 187:191–9. 10.1002/(SICI)1096-9896(199901)187:2<191::AID-PATH206>3.0.CO;2-3 10365094

[193] van der Stoep N Biesta P Quinten E van den Elsen PJ. Lack of IFN-gamma-mediated induction of the class II transactivator (CIITA) through promoter methylation is predominantly found in developmental tumor cell lines. Int J Cancer (2002) 97:501–7. 10.1002/ijc.1623 11802213

[194] Croce M De Ambrosis A Corrias MV Pistoia V Occhino M Meazza R Different levels of control prevent interferon-gamma-inducible HLA-class II expression in human neuroblastoma cells. Oncogene (2003) 22:7848–57. 10.1038/sj.onc.1207054 14586411

[195] Dunn GP Koebel CM Schreiber RD. Interferons, immunity and cancer immunoediting. Nat Rev Immunol (2006) 6:836–48. 10.1038/nri1961 17063185

[196] Dunn GP Ikeda H Bruce AT Koebel C Uppaluri R Bui J Interferon-gamma and cancer immunoediting. Immunol Res (2005) 32:231–45. 10.1385/IR:32:1-3:231 16106075

[197] Bromberg JF Horvath CM Wen Z Schreiber RD Darnell JE Jr. Transcriptionally active Stat1 is required for the antiproliferative effects of both interferon alpha and interferon gamma. Proc Natl Acad Sci U S A (1996) 93:7673–8. 10.1073/pnas.93.15.7673 8755534 PMC38805

[198] Fulda S Debatin KM. IFNgamma sensitizes for apoptosis by upregulating caspase-8 expression through the Stat1 pathway. Oncogene (2002) 21:2295–308. 10.1038/sj.onc.1205255 11948413

[199] Chin YE Kitagawa M Kuida K Flavell RA Fu XY. Activation of the STAT signaling pathway can cause expression of caspase 1 and apoptosis. Mol Cell Biol (1997) 17:5328–37. 10.1128/MCB.17.9.5328 9271410 PMC232383

[200] Xu X Fu XY Plate J Chong AS. IFN-gamma induces cell growth inhibition by Fas-mediated apoptosis: requirement of STAT1 protein for up-regulation of Fas and FasL expression. Cancer Res (1998) 58:2832–7. 9661898

[201] Liu F Hu X Zimmerman M Waller JL Wu P Hayes-Jordan A TNFalpha cooperates with IFN-gamma to repress Bcl-xL expression to sensitize metastatic colon carcinoma cells to TRAIL-mediated apoptosis. PLoS One (2011) 6:e16241 10.1371/journal.pone.0016241 21264227 PMC3022032

[202] Takeda K Smyth MJ Cretney E Hayakawa Y Kayagaki N Yagita H Critical role for tumor necrosis factor-related apoptosis-inducing ligand in immune surveillance against tumor development. J Exp Med (2002) 195:161–9. 10.1084/jem.20011171 11805143 PMC2193611

[203] Thapa RJ Basagoudanavar SH Nogusa S Irrinki K Mallilankaraman K Slifker MJ NF-kappaB protects cells from gamma interferon-induced RIP1-dependent necroptosis. Mol Cell Biol (2011) 31:2934–46. 10.1128/MCB.05445-11 21576359 PMC3133390

[204] Hayakawa Y Takeda K Yagita H Smyth MJ Van Kaer L Okumura K IFN-gamma-mediated inhibition of tumor angiogenesis by natural killer T-cell ligand, alpha-galactosylceramide. Blood (2002) 100:1728–33. 12176894

[205] Kammertoens T Friese C Arina A Idel C Briesemeister D Rothe M Tumour ischaemia by interferon-gamma resembles physiological blood vessel regression. Nature (2017) 545:98–102. 10.1038/nature22311 28445461 PMC5567674

[206] Briesemeister D Sommermeyer D Loddenkemper C Loew R Uckert W Blankenstein T Tumor rejection by local interferon gamma induction in established tumors is associated with blood vessel destruction and necrosis. Int J Cancer (2011) 128:371–8. 10.1002/ijc.25350 20333679

[207] Martini M Testi MG Pasetto M Picchio MC Innamorati G Mazzocco M IFN-gamma-mediated upmodulation of MHC class I expression activates tumor-specific immune response in a mouse model of prostate cancer. Vaccine (2010) 28:3548–57. 10.1016/j.vaccine.2010.03.007 20304037

[208] Groom JR Luster AD. CXCR3 ligands: redundant, collaborative and antagonistic functions. Immunol Cell Biol (2011) 89:207–15. 10.1038/icb.2010.158 21221121 PMC3863330

[209] Melero I Rouzaut A Motz GT Coukos G. T-cell and NK-cell infiltration into solid tumors: a key limiting factor for efficacious cancer immunotherapy. Cancer Discov (2014) 4:522–6. 10.1158/2159-8290.CD-13-0985 24795012 PMC4142435

[210] Barreira da Silva R Laird ME Yatim N Fiette L Ingersoll MA Albert ML. Dipeptidylpeptidase 4 inhibition enhances lymphocyte trafficking, improving both naturally occurring tumor immunity and immunotherapy. Nat Immunol (2015) 16:850–8. 10.1038/ni.3201 26075911

[211] Gordon-Alonso M Hirsch T Wildmann C van der Bruggen P. Galectin-3 captures interferon-gamma in the tumor matrix reducing chemokine gradient production and T-cell tumor infiltration. Nat Commun (2017) 8:793. 10.1038/s41467-017-00925-6 28986561 PMC5630615

[212] Campanella GS Colvin RA Luster AD. CXCL10 can inhibit endothelial cell proliferation independently of CXCR3. PLoS One (2010) 5:e12700. 10.1371/journal.pone.0012700 20856926 PMC2938333

[213] Feldman ED Weinreich DM Carroll NM Burness ML Feldman AL Turner E Interferon gamma-inducible protein 10 selectively inhibits proliferation and induces apoptosis in endothelial cells. Ann Surg Oncol (2006) 13:125–33. 10.1245/ASO.2006.03.038 16378159

[214] Zhang T Sun HC Zhou HY Luo JT Zhang BL Wang P Interferon alpha inhibits hepatocellular carcinoma growth through inducing apoptosis and interfering with adhesion of tumor endothelial cells. Cancer Lett (2010) 290:204–10. 10.1016/j.canlet.2009.09.009 19822391

[215] Ligocki AJ Brown JR Niederkorn JY Role of interferon-gamma and cytotoxic T lymphocytes in intraocular tumor rejection. J Leukoc Biol (2016) 99:735–47. 10.1189/jlb.3A0315-093RRR 26578649 PMC4831480

[216] Zimmerman M Yang D Hu X Liu F Singh N Browning D IFN-gamma upregulates survivin and Ifi202 expression to induce survival and proliferation of tumor-specific T cells. PLoS One (2010) 5:e14076 10.1371/journal.pone.0014076 21124930 PMC2989915

[217] Celada A Gray PW Rinderknecht E Schreiber RD. Evidence for a gamma-interferon receptor that regulates macrophage tumoricidal activity. J Exp Med (1984) 160:55–74. 10.1084/jem.160.1.55 6330272 PMC2187421

[218] Sinha P Clements VK Ostrand-Rosenberg S. Reduction of myeloid-derived suppressor cells and induction of M1 macrophages facilitate the rejection of established metastatic disease. J Immunol (2005) 174:636–45. 10.4049/jimmunol.174.2.636 15634881

[219] Zhang C Hou D Wei H Zhao M Yang L Liu Q Lack of interferon-gamma receptor results in a microenvironment favorable for intestinal tumorigenesis. Oncotarget (2016) 7:42099–109. 10.18632/oncotarget.9867 27286456 PMC5173119

[220] Duluc D Corvaisier M Blanchard S Catala L Descamps P Gamelin E Interferon-gamma reverses the immunosuppressive and protumoral properties and prevents the generation of human tumor-associated macrophages. Int J Cancer (2009) 125:367–73. 10.1002/ijc.24401 19378341

[221] Cardoso AP Goncalves RM Antunes JC Pinto ML Pinto AT Castro F An interferon-gamma-delivery system based on chitosan/poly(gamma-glutamic acid) polyelectrolyte complexes modulates macrophage-derived stimulation of cancer cell invasion in vitro. Acta Biomater (2015) 23:157–71. 10.1016/j.actbio.2015.05.022 26013040

[222] Yu WG Ogawa M Mu J Umehara K Tsujimura T Fujiwara H IL-12-induced tumor regression correlates with in situ activity of IFN-gamma produced by tumor-infiltrating cells and its secondary induction of anti-tumor pathways. J Leukoc Biol (1997) 62:450–7. 10.1002/jlb.62.4.450 9335314

[223] Coughlin CM Salhany KE Gee MS LaTemple DC Kotenko S Ma X Tumor cell responses to IFNgamma affect tumorigenicity and response to IL-12 therapy and antiangiogenesis. Immunity (1998) 9:25–34. 10.1016/S1074-7613(00)80585-3 9697833

[224] Abdi K Singh N Matzinger P. T-cell control of IL-12p75 production. Scand J Immunol (2006) 64:83–92. 10.1111/j.1365-3083.2006.01767.x 16867152

[225] Snijders A Kalinski P Hilkens CM Kapsenberg ML. High-level IL-12 production by human dendritic cells requires two signals. Int Immunol (1998) 10:1593–8. 10.1093/intimm/10.11.1593 9846688

[226] Noguchi Y Jungbluth A Richards EC Old LJ. Effect of interleukin 12 on tumor induction by 3-methylcholanthrene. Proc Natl Acad Sci U S A (1996) 93:11798–801. 10.1073/pnas.93.21.11798 8876217 PMC38138

[227] Komita H Homma S Saotome H Zeniya M Ohno T Toda G. Interferon-gamma produced by interleukin-12-activated tumor infiltrating CD8+T cells directly induces apoptosis of mouse hepatocellular carcinoma. J Hepatol (2006) 45:662–72. 10.1016/j.jhep.2006.05.018 16935390

[228] Chmielewski M Kopecky C Hombach AA Abken H. IL-12 release by engineered T cells expressing chimeric antigen receptors can effectively Muster an antigen-independent macrophage response on tumor cells that have shut down tumor antigen expression. Cancer Res (2011) 71:5697–706. 10.1158/0008-5472.CAN-11-0103 21742772

[229] Pegram HJ Lee JC Hayman EG Imperato GH Tedder TF Sadelain M Tumor-targeted T cells modified to secrete IL-12 eradicate systemic tumors without need for prior conditioning. Blood (2012) 119:4133–41. 10.1182/blood-2011-12-400044 22354001 PMC3359735

[230] Zhang L Kerkar SP Yu Z Zheng Z Yang S Restifo NP Improving adoptive T cell therapy by targeting and controlling IL-12 expression to the tumor environment. Mol Ther (2011) 19:751–9. 10.1038/mt.2010.313 21285960 PMC3070103

[231] Simpson JA Al-Attar A Watson NF Scholefield JH Ilyas M Durrant LG. Intratumoral T cell infiltration, MHC class I and STAT1 as biomarkers of good prognosis in colorectal cancer. Gut (2010) 59:926–33. 10.1136/gut.2009.194472 20581241

[232] Fridman WH Pages F Sautes-Fridman C Galon J. The immune contexture in human tumours: impact on clinical outcome. Nat Rev Cancer (2012) 12:298–306. 10.1038/nrc3245 22419253

[233] Ayers M Lunceford J Nebozhyn M Murphy E Loboda A Kaufman DR IFN-gamma-related mRNA profile predicts clinical response to PD-1 blockade. J Clin Invest (2017) 127:2930–40. 10.1172/JCI91190 28650338 PMC5531419

[234] Karachaliou N Gonzalez-Cao M Crespo G Drozdowskyj A Aldeguer E Gimenez-Capitan A Interferon gamma, an important marker of response to immune checkpoint blockade in non-small cell lung cancer and melanoma patients. Ther Adv Med Oncol (2018) 10:1758834017749748. 10.1177/1758834017749748 29383037 PMC5784541

[235] Mo X Zhang H Preston S Martin K Zhou B Vadalia N Interferon-gamma signaling in melanocytes and melanoma cells regulates expression of CTLA-4. Cancer Res (2018) 78:436–50. 10.1158/0008-5472.CAN-17-1615 29150430 PMC5771950

[236] Curran MA Montalvo W Yagita H Allison JP. PD-1 and CTLA-4 combination blockade expands infiltrating T cells and reduces regulatory T and myeloid cells within B16 melanoma tumors. Proc Natl Acad Sci U S A (2010) 107:4275–80. 10.1073/pnas.0915174107 20160101 PMC2840093

[237] Curran MA Kim M Montalvo W Al-Shamkhani A Allison JP. Combination CTLA-4 blockade and 4-1BB activation enhances tumor rejection by increasing T-cell infiltration, proliferation, and cytokine production. PLoS One (2011) 6:e19499. 10.1371/journal.pone.0019499 21559358 PMC3085474

[238] Liakou CI Kamat A Tang DN Chen H Sun J Troncoso P CTLA-4 blockade increases IFNgamma-producing CD4+ICOShi cells to shift the ratio of effector to regulatory T cells in cancer patients. Proc Natl Acad Sci U S A (2008) 105:14987–92. 10.1073/pnas.0806075105 18818309 PMC2567480

[239] Peng W Liu C Xu C Lou Y Chen J Yang Y PD-1 blockade enhances T-cell migration to tumors by elevating IFN-gamma inducible chemokines. Cancer Res (2012) 72:5209–18. 10.1158/0008-5472.CAN-12-1187 22915761 PMC3476734

[240] Overacre-Delgoffe AE Chikina M Dadey RE Yano H Brunazzi EA Shayan G Interferon-gamma drives Treg fragility to promote anti-tumor immunity. Cell (2017) 169:1130–41.e11. 10.1016/j.cell.2017.05.005 28552348 PMC5509332

[241] Gao J Shi LZ Zhao H Chen J Xiong L He Q Loss of IFN-gamma pathway genes in tumor cells as a mechanism of resistance to anti-CTLA-4 therapy. Cell (2016) 167:397–404.e9. 10.1016/j.cell.2016.08.069 27667683 PMC5088716

[242] Patel SJ Sanjana NE Kishton RJ Eidizadeh A Vodnala SK Cam M Identification of essential genes for cancer immunotherapy. Nature (2017) 548:537–42. 10.1038/nature23477 28783722 PMC5870757

[243] Windbichler GH Hausmaninger H Stummvoll W Graf AH Kainz C Lahodny J Interferon-gamma in the first-line therapy of ovarian cancer: a randomized phase III trial. Br J Cancer (2000) 82:1138–44. 10.1054/bjoc.1999.1053 10735496 PMC2363351

[244] Giannopoulos A Constantinides C Fokaeas E Stravodimos C Giannopoulou M Kyroudi A The immunomodulating effect of interferon-gamma intravesical instillations in preventing bladder cancer recurrence. Clin Cancer Res (2003) 9:5550–8. 14654535

[245] Khammari A Nguyen JM Saint-Jean M Knol AC Pandolfino MC Quereux G Adoptive T cell therapy combined with intralesional administrations of TG1042 (adenovirus expressing interferon-gamma) in metastatic melanoma patients. Cancer Immunol Immunother (2015) 64:805–15. 10.1007/s00262-015-1691-7 25846669 PMC11029588

[246] Lesinski GB Zimmerer JM Kreiner M Trefry J Bill MA Young GS Modulation of SOCS protein expression influences the interferon responsiveness of human melanoma cells. BMC Cancer (2010) 10:142. 10.1186/1471-2407-10-142 20398276 PMC2858748

[247] Evans MK Yu CR Lohani A Mahdi RM Liu X Trzeciak AR Expression of SOCS1 and SOCS3 genes is differentially regulated in breast cancer cells in response to proinflammatory cytokine and growth factor signals. Oncogene (2007) 26:1941–8. 10.1038/sj.onc.1209993 17001312

[248] Zaidi MR Merlino G The two faces of interferon-gamma in cancer. Clin Cancer Res (2011) 17:6118–24. 10.1158/1078-0432.CCR-11-0482 21705455 PMC3186825

[249] Taniguchi K Petersson M Hoglund P Kiessling R Klein G Karre K. Interferon gamma induces lung colonization by intravenously inoculated B16 melanoma cells in parallel with enhanced expression of class I major histocompatibility complex antigens. Proc Natl Acad Sci U S A (1987) 84:3405–9. 10.1073/pnas.84.10.3405 3106968 PMC304879

[250] Beatty GL Paterson Y IFN-gamma can promote tumor evasion of the immune system in vivo by down-regulating cellular levels of an endogenous tumor antigen. J Immunol (2000) 165:5502–8. 10.4049/jimmunol.165.10.5502 11067903

[251] Brocker EB Zwadlo G Holzmann B Macher E Sorg C. Inflammatory cell infiltrates in human melanoma at different stages of tumor progression. Int J Cancer (1988) 41:562–7. 10.1002/ijc.2910410415 3128489

[252] Garbe C Krasagakis K Zouboulis CC Schroder K Kruger S Stadler R Antitumor activities of interferon alpha, beta, and gamma and their combinations on human melanoma cells in vitro: changes of proliferation, melanin synthesis, and immunophenotype. J Invest Dermatol (1990) 95:231S–7S. 10.1111/1523-1747.ep12875837 2124247

[253] Brody JR Costantino CL Berger AC Sato T Lisanti MP Yeo CJ Expression of indoleamine 2,3-dioxygenase in metastatic malignant melanoma recruits regulatory T cells to avoid immune detection and affects survival. Cell Cycle (2009) 8:1930–4. 10.4161/cc.8.12.8745 19448397

[254] Cripps JG Wang J Maria A Blumenthal I Gorham JD. Type 1 T helper cells induce the accumulation of myeloid-derived suppressor cells in the inflamed Tgfb1 knockout mouse liver. Hepatology (2010) 52:1350–9. 10.1002/hep.23841 20803559 PMC2947571

[255] Mundy-Bosse BL Lesinski GB Jaime-Ramirez AC Benninger K Khan M Kuppusamy P Myeloid-derived suppressor cell inhibition of the IFN response in tumor-bearing mice. Cancer Res (2011) 71:5101–10. 10.1158/0008-5472.CAN-10-2670 21680779 PMC3148319

[256] Abiko K Mandai M Hamanishi J Yoshioka Y Matsumura N Baba T PD-L1 on tumor cells is induced in ascites and promotes peritoneal dissemination of ovarian cancer through CTL dysfunction. Clin Cancer Res (2013) 19:1363–74. 10.1158/1078-0432.CCR-12-2199 23340297

[257] Zaretsky JM Garcia-Diaz A Shin DS Escuin-Ordinas H Hugo W Hu-Lieskovan S Mutations associated with acquired resistance to PD-1 blockade in melanoma. N Engl J Med (2016) 375:819–29. 10.1056/NEJMoa1604958 27433843 PMC5007206

[258] Shin DS Zaretsky JM Escuin-Ordinas H Garcia-Diaz A Hu-Lieskovan S Kalbasi A Primary resistance to PD-1 blockade mediated by JAK1/2 mutations. Cancer Discov (2017) 7:188–201. 10.1158/2159-8290.CD-16-1223 27903500 PMC5296316

[259] Benci JL Xu B Qiu Y Wu TJ Dada H Twyman-Saint Victor C Tumor interferon signaling regulates a multigenic resistance program to immune checkpoint blockade. Cell (2016) 167:1540–54.e12. 10.1016/j.cell.2016.11.022 27912061 PMC5385895

[260] Wang XB Zheng CY Giscombe R Lefvert AK. Regulation of surface and intracellular expression of CTLA-4 on human peripheral T cells. Scand J Immunol (2001) 54:453–8. 10.1046/j.1365-3083.2001.00985.x 11696196

[261] Creagan ET Ahmann DL Long HJ Frytak S Sherwin SA Chang MN. Phase II study of recombinant interferon-gamma in patients with disseminated malignant melanoma. Cancer Treat Rep (1987) 71:843–4. 3113730

[262] Ernstoff MS Trautman T Davis CA Reich SD Witman P Balser J A randomized phase I/II study of continuous versus intermittent intravenous interferon gamma in patients with metastatic melanoma. J Clin Oncol (1987) 5:1804–10. 10.1200/JCO.1987.5.11.1804 3119786

[263] Kopp WC Smith JW II Ewel CH Alvord WG Main C Guyre PM Immunomodulatory effects of interferon-gamma in patients with metastatic malignant melanoma. J Immunother Emphasis Tumor Immunol (1993) 13:181–90. 10.1097/00002371-199304000-00005 8471592

[264] Khorana AA Rosenblatt JD Sahasrabudhe DM Evans T Ladrigan M Marquis D A phase I trial of immunotherapy with intratumoral adenovirus-interferon-gamma (TG1041) in patients with malignant melanoma. Cancer Gene Ther (2003) 10:251–9. 10.1038/sj.cgt.7700568 12679797

[265] Gleave ME Elhilali M Fradet Y Davis I Venner P Saad F Interferon gamma-1b compared with placebo in metastatic renal-cell carcinoma. Canadian Urologic Oncology Group. N Engl J Med (1998) 338:1265–71. 10.1056/NEJM199804303381804 9562580

[266] Talpaz M Kurzrock R Kantarjian H Rothberg J Saks S Evans L A phase II study alternating alpha-2a-interferon and gamma-interferon therapy in patients with chronic myelogenous leukemia. Cancer (1991) 68:2125–30. 10.1002/1097-0142(19911115)68:10<2125::AID-CNCR2820681006>3.0.CO;2-Q 1913450

[267] Von Hoff DD Fleming TR Macdonald JS Goodman PJ Van Damme J Brown TD Phase II evaluation of recombinant gamma-interferon in patients with advanced pancreatic carcinoma: a Southwest Oncology Group study. J Biol Response Mod (1990) 9:584–7. 2127424

[268] Vahdat LT Cohen DJ Zipin D Lo KS Donovan D Savage D Randomized trial of low-dose interleukin-2 vs cyclosporine A and interferon-gamma after high-dose chemotherapy with peripheral blood progenitor support in women with high-risk primary breast cancer. Bone Marrow Transplant (2007) 40:267–72. 10.1038/sj.bmt.1705692 17563739

[269] Wiesenfeld M O’Connell MJ Wieand HS Gonchoroff NJ Donohue JH Fitzgibbons RJ Jr Controlled clinical trial of interferon-gamma as postoperative surgical adjuvant therapy for colon cancer. J Clin Oncol (1995) 13:2324–9. 10.1200/JCO.1995.13.9.2324 7666090

[270] Alberts DS Marth C Alvarez RD Johnson G Bidzinski M Kardatzke DR Randomized phase 3 trial of interferon gamma-1b plus standard carboplatin/paclitaxel versus carboplatin/paclitaxel alone for first-line treatment of advanced ovarian and primary peritoneal carcinomas: results from a prospectively designed analysis of progression-free survival. Gynecol Oncol (2008) 109:174–81. 10.1016/j.ygyno.2008.01.005 18314182

[271] Kim OY Park HT Dinh NTH Choi SJ Lee J Kim JH Bacterial outer membrane vesicles suppress tumor by interferon-gamma-mediated antitumor response. Nat Commun (2017) 8:626 10.1038/s41467-017-00729-8 28931823 PMC5606984

[272] Kim JS Park YM Kim NY Yun HK Lee KJ Kim BH Combination treatment with intrahepatic arterial infusion and intratumoral injection chemotherapy in patients with far-advanced hepatocellular carcinoma and arterioportal or arteriovenous shunts: preliminary results. Korean J Hepatol (2011) 17:120–9. 10.3350/kjhep.2011.17.2.120 21757983 PMC3304631

[273] Fridman WH Zitvogel L Sautes-Fridman C Kroemer G. The immune contexture in cancer prognosis and treatment. Nat Rev Clin Oncol (2017) 14(12):717–34. 10.1038/nrclinonc.2017 28741618

[274] Estrov ZKR Talpaz M Interferons: Basic Principles and Clinical Applications. Portland: Book News, Inc. (1993).

[275] Razaghi A Owens L Heimann K. Review of the recombinant human interferon gamma as an immunotherapeutic: impacts of production platforms and glycosylation. J Biotechnol (2016) 240:48–60. 10.1016/j.jbiotec.2016.10.022 27794496

[276] Kurzrock R Rosenblum MG Sherwin SA Rios A Talpaz M Quesada JR Pharmacokinetics, single-dose tolerance, and biological activity of recombinant gamma-interferon in cancer patients. Cancer Res (1985) 45:2866–72. 3921249

[277] Devane JG Martin ML Matson MA. A short 2 week dose titration regimen reduces the severity of flu-like symptoms with initial interferon gamma-1b treatment. Curr Med Res Opin (2014) 30:1179–87. 10.1185/03007995.2014.899209 24576196

[278] van Loon AP Ozmen L Fountoulakis M Kania M Haiker M Garotta G. High-affinity receptor for interferon-gamma (IFN-gamma), a ubiquitous protein occurring in different molecular forms on human cells: blood monocytes and eleven different cell lines have the same IFN-gamma receptor protein. J Leukoc Biol (1991) 49:462–73. 10.1002/jlb.49.5.462 1826725

[279] Valente G Ozmen L Novelli F Geuna M Palestro G Forni G Distribution of interferon-gamma receptor in human tissues. Eur J Immunol (1992) 22:2403–12. 10.1002/eji.1830220933 1387613

[280] Bello I Perez A Torres AM Hernandez MV Lopez Saura P. High levels of soluble IFN gamma receptor alpha chain in the plasma of rheumatoid arthritis patients. Biotherapy (1998) 11:53–7. 10.1023/A:1008087100315 9617465

[281] Nathan CF Kaplan G Levis WR Nusrat A Witmer MD Sherwin SA Local and systemic effects of intradermal recombinant interferon-gamma in patients with lepromatous leprosy. N Engl J Med (1986) 315:6–15. 10.1056/NEJM198607033150102 3086725

[282] Edwards L Whiting D Rogers D Luck K Smiles KA. The effect of intralesional interferon gamma on basal cell carcinomas. J Am Acad Dermatol (1990) 22:496–500. 10.1016/0190-9622(90)70070-X 2107219

[283] Thom AK Fraker DL Taubenberger JK Norton JA. Effective regional therapy of experimental cancer with paralesional administration of tumour necrosis factor-alpha + interferon-gamma. Surg Oncol (1992) 1:291–8. 10.1016/0960-7404(92)90090-8 1341263

[284] Lejeune F Lienard D Eggermont A Schraffordt Koops H Rosenkaimer F Gerain J Rationale for using TNF alpha and chemotherapy in regional therapy of melanoma. J Cell Biochem (1994) 56:52–61. 10.1002/jcb.240560110 7806592

[285] Sokoloff MH Tso CL Kaboo R Taneja S Pang S deKernion JB In vitro modulation of tumor progression-associated properties of hormone refractory prostate carcinoma cell lines by cytokines. Cancer (1996) 77:1862–72. 10.1002/(SICI)1097-0142(19960501)77:9<1862::AID-CNCR16>3.0.CO;2-Y 8646686

[286] Habif DV Ozzello L De Rosa CM Cantell K Lattes R. Regression of skin recurrences of breast carcinomas treated with intralesional injections of natural interferons alpha and gamma. Cancer Invest (1995) 13:165–72. 10.3109/07357909509011686 7874570

[287] Weiss JM Muchenberger S Schopf E Simon JC Treatment of granuloma annulare by local injections with low-dose recombinant human interferon gamma. J Am Acad Dermatol (1998) 39:117–9. 10.1016/S0190-9622(98)70412-8 9674407

[288] Bocci V Roles of interferon produced in physiological conditions. A speculative review. Immunology (1988) 64:1–9. 2454881 PMC1385178

[289] Younes HM Amsden BG. Interferon-gamma therapy: evaluation of routes of administration and delivery systems. J Pharm Sci (2002) 91:2–17. 10.1002/jps.10007 11782893

[290] Hayakawa M Hatano T Miyazato T Sato K Saito S Osawa A [Studies on the antitumor effects of human interferon (2). Effects of massive pulsatile administration of rIFN-gamma on advanced renal cell carcinoma]. Nihon Hinyokika Gakkai Zasshi (1987) 78:1784–91. 3127620 10.5980/jpnjurol1928.78.10_1784

[291] Murray HW. Effect of continuous administration of interferon-gamma in experimental visceral leishmaniasis. J Infect Dis (1990) 161:992–4. 10.1093/infdis/161.5.992 2157773

[292] Cantell K Hirvonen S Pyhala L De Reus A Schellekens H. Circulating interferon in rabbits and monkeys after administration of human gamma interferon by different routes. J Gen Virol (1983) 64(Pt 8):1823–6. 10.1099/0022-1317-64-8-1823 6409993

[293] Levine RU Crum CP Herman E Silvers D Ferenczy A Richart RM. Cervical papillomavirus infection and intraepithelial neoplasia: a study of male sexual partners. Obstet Gynecol (1984) 64:16–20. 6330630

[294] Gross G Roussaki A Papendick U. Efficacy of interferons on bowenoid papulosis and other precancerous lesions. J Invest Dermatol (1990) 95:152S–7S. 10.1111/1523-1747.ep12875145 1701802

[295] Hautmann SH Huland E Huland H. Local intratumor immunotherapy of prostate cancer with interleukin-2 reduces tumor growth. Anticancer Res (1999) 19:2661–3. 10470215

[296] Zhao Z Leong KW. Controlled delivery of antigens and adjuvants in vaccine development. J Pharm Sci (1996) 85:1261–70. 10.1021/js9602812 8961136

[297] van Slooten ML Storm G Zoephel A Kupcu Z Boerman O Crommelin DJ Liposomes containing interferon-gamma as adjuvant in tumor cell vaccines. Pharm Res (2000) 17:42–8. 10.1023/A:1007514424253 10714607

[298] Mejias R Perez-Yague S Gutierrez L Cabrera LI Spada R Acedo P Dimercaptosuccinic acid-coated magnetite nanoparticles for magnetically guided in vivo delivery of interferon gamma for cancer immunotherapy. Biomaterials (2011) 32:2938–52. 10.1016/j.biomaterials.2011.01.008 21277630

[299] Segura S Gamazo C Irache JM Espuelas S. Gamma interferon loaded onto albumin nanoparticles: in vitro and in vivo activities against *Brucella abortus*. Antimicrob Agents Chemother (2007) 51:1310–4. 10.1128/AAC.00890-06 17220401 PMC1855480

[300] Ando M Takahashi Y Yamashita T Fujimoto M Nishikawa M Watanabe Y Prevention of adverse events of interferon gamma gene therapy by gene delivery of interferon gamma-heparin-binding domain fusion protein in mice. Mol Ther Methods Clin Dev (2014) 1:14023 10.1038/mtm.2014.23 26015966 PMC4362348

[301] Badea I Virtanen C Verrall RE Rosenberg A Foldvari M Effect of topical interferon-gamma gene therapy using gemini nanoparticles on pathophysiological markers of cutaneous scleroderma in Tsk/+ mice. Gene Ther (2012) 19:978–87. 10.1038/gt.2011.159 22071972

[302] Bourgeois-Daigneault MC Roy DG Falls T Twumasi-Boateng K St-Germain LE Marguerie M Oncolytic vesicular stomatitis virus expressing interferon-gamma has enhanced therapeutic activity. Mol Ther Oncolytics (2016) 3:16001 10.1038/mto.2016.1 27119116 PMC4824565

[303] Zanganeh S Hutter G Spitler R Lenkov O Mahmoudi M Shaw A Iron oxide nanoparticles inhibit tumour growth by inducing pro-inflammatory macrophage polarization in tumour tissues. Nat Nanotechnol (2016) 11:986–94. 10.1038/nnano.2016.168 27668795 PMC5198777

[304] van Broekhoven CL Parish CR Demangel C Britton WJ Altin JG. Targe-ting dendritic cells with antigen-containing liposomes: a highly effective procedure for induction of antitumor immunity and for tumor immunotherapy. Cancer Res (2004) 64:4357–65. 10.1158/0008-5472.CAN-04-0138 15205352

[305] Cruz LJ Rosalia RA Kleinovink JW Rueda F Lowik CW Ossendorp F. Targeting nanoparticles to CD40, DEC-205 or CD11c molecules on dendritic cells for efficient CD8(+) T cell response: a comparative study. J Control Release (2014) 192:209–18. 10.1016/j.jconrel.2014.07.040 25068703

[306] Castro F Pinto ML Silva AM Pereira CL Teixeira GQ Gomez-Lazaro M Pro-inflammatory chitosan/poly(gamma-glutamic acid) nanoparticles modulate human antigen-presenting cells phenotype and revert their pro-invasive capacity. Acta Biomater (2017) 63:96–109. 10.1016/j.actbio.2017.09.016 28919508

[307] Kumar M Coburn J Kaplan DL Mandal BB. Immuno-informed 3D silk biomaterials for tailoring biological responses. ACS Appl Mater Interfaces (2016) 8:29310–22. 10.1021/acsami.6b09937 27726371

[308] Ebbinghaus C Ronca R Kaspar M Grabulovski D Berndt A Kosmehl H Engineered vascular-targeting antibody-interferon-gamma fusion protein for cancer therapy. Int J Cancer (2005) 116:304–13. 10.1002/ijc.20952 15800913

